# Counteracting neuroinflammation in experimental Parkinson’s disease favors recovery of function: effects of Er-NPCs administration

**DOI:** 10.1186/s12974-018-1375-2

**Published:** 2018-11-30

**Authors:** Stephana Carelli, Toniella Giallongo, Zuzana Gombalova, Federica Rey, Maria Carlotta F. Gorio, Massimiliano Mazza, Anna Maria Di Giulio

**Affiliations:** 1 Laboratory of Pharmacology, Department of Health Sciences, University of Milan, Polo H. San Paolo, via A di Rudinì 8, 20142 Milan, Italy; 2 Pediatric Clinical Research Center Fondazione Romeo e Enrica Invernizzi, University of Milan, Milan, Italy; 3 Faculty of Science, Institute of Biology and Ecology, Pavol Jozef Safarik University in Kosice, Moyzesova 11, 04001 Kosice, Slovakia; 4 Department of Biomedical and Clinical Science L. Sacco, University of Milan, Milan, Italy; 5 Istituto Scientifico Romagnolo per lo Studio e la Cura dei Tumori (IRST) IRCCS, Via Piero Maroncelli 40, 47014 Meldola, FC Italy

**Keywords:** Parkinson’s disease, Erythropoietin, Adult stem cells, Neural stem cells transplantation, Neuroinflammation, Regenerative medicine

## Abstract

**Background:**

Parkinson’s disease (PD) is the second most common neurodegenerative disease, presenting with midbrain dopaminergic neurons degeneration. A number of studies suggest that microglial activation may have a role in PD. It has emerged that inflammation-derived oxidative stress and cytokine-dependent toxicity may contribute to nigrostriatal pathway degeneration and exacerbate the progression of the disease in patients with idiopathic PD. Cell therapies have long been considered a feasible regenerative approach to compensate for the loss of specific cell populations such as the one that occurs in PD. We recently demonstrated that erythropoietin-releasing neural precursors cells (Er-NPCs) administered to MPTP-intoxicated animals survive after transplantation in the recipient’s damaged brain, differentiate, and rescue degenerating striatal dopaminergic neurons. Here, we aimed to investigate the potential anti-inflammatory actions of Er-NPCs infused in an MPTP experimental model of PD.

**Methods:**

The degeneration of dopaminergic neurons was caused by MPTP administration in C57BL/6 male mice. 2.5 × 10^5^ GFP-labeled Er-NPCs were administered by stereotaxic injection unilaterally in the left striatum. Functional recovery was assessed by two independent behavioral tests. Neuroinflammation was investigated measuring the mRNAs levels of pro-inflammatory and anti-inflammatory cytokines, and immunohistochemistry studies were performed to evaluate markers of inflammation and the potential rescue of tyrosine hydroxylase (TH) projections in the striatum of recipient mice.

**Results:**

Er-NPC administration promoted a rapid anti-inflammatory effect that was already evident 24 h after transplant with a decrease of pro-inflammatory and increase of anti-inflammatory cytokines mRNA expression levels. This effect was maintained until the end of the observational period, 2 weeks post-transplant. Here, we show that Er-NPCs transplant reduces macrophage infiltration, directly counteracting the M1-like pro-inflammatory response of murine-activated microglia, which corresponds to the decrease of CD68 and CD86 markers, and induces M2-like pro-regeneration traits, as indicated by the increase of CD206 and IL-10 expression. Moreover, we also show that this activity is mediated by Er-NPCs-derived erythropoietin (EPO) since the co-injection of cells with anti-EPO antibodies neutralizes the anti-inflammatory effect of the Er-NPCs treatment.

**Conclusion:**

This study shows the anti-inflammatory actions exerted by Er-NPCs, and we suggest that these cells may represent good candidates for cellular therapy to counteract neuroinflammation in neurodegenerative disorders.

**Electronic supplementary material:**

The online version of this article (10.1186/s12974-018-1375-2) contains supplementary material, which is available to authorized users.

## Background

Parkinson’s disease (PD) is characterized by dopaminergic (DA) denervation of the striatum and progressive death of DA neurons in the substantia nigra pars compacta (SNpc) [1]. Neuroinflammation has a role in several neurodegenerative diseases; though it may not be considered the primary cause, it contributes to the symptomatic phase [2]. Several lines of research suggest that neuroinflammation is the major central event in dopaminergic neural cell death in PD [3–5]. In postmortem SN from human PD brains, microglia results activated, lymphocytes are infiltrated [2, 6], and in cerebrospinal fluids, there are high levels of pro-inflammatory cytokines such as tumor necrosis factor (TNF), cyclooxygenase-2 (COX-2), interleukin-1beta (IL-1beta), and IL-18 [7, 8]. It has been suggested that in CNS neurodegeneration, neuronal damage may lead to the activation of microglia and astrocytes that in turn amplify the inflammatory response through chemokine secretion. This enhances CNS infiltration by peripheral immune cells. Studies in the acute neurotoxic 1-methyl-4-phenyl-1,2,3,6-tetrahydropyridine (MPTP) mouse model provided evidences that neuroinflammatory processes can contribute to nigral DA neuronal death [9–11]. Both the genetic deletions of microglial effectors [12] and the suppression of T lymphocytes [6] reduced neuronal loss suggesting that neuroinflammation in PD may actively participate in neuronal death. The “principal mechanism” that links the inflammatory response with neurodegeneration remains to be fully clarified. However, it is recognized that neuronal degeneration itself and in particular the accumulation and release of alpha-synuclein aggregates by the injured DA neurons early in the disease process [13], may act as a signal, and can activate glial cells to produce and release a variety of pro-inflammatory molecules, exacerbating microglia activation and neuronal cell death [14–18]. Within this scenario, the major players are the microglia, the reactive astrocytes, and the infiltrating monocyte-derived macrophages [19]. Upon injury, activated M1-like microglia proliferates and participates in clearing cell debris in the early stages but may exacerbate brain injury through the production of neurotoxic substances, especially when it is overactivated for prolonged periods [20]. In the M2-like phenotype, microglia has anti-inflammatory and neuron-reparative roles, protecting the damaged tissue by removing cell debris and releasing anti-inflammatory cytokines needed for tissue repair [21]. Current treatments for PD are only symptomatic and have no effects on the ongoing neurodegeneration [22]. The ideal therapeutic treatment for PD should have both symptomatic and restorative effects aimed at preserving midbrain DA neurons from degeneration [23, 24]. In this sense, adult neural stem cells grafting into animal experimental models of neurodegenerative diseases have shown beneficial effects promoting both trophic and anti-inflammatory actions [25–31]. Recently, we reported the therapeutic potential of erythropoietin-releasing neural precursors cells (Er-NPCs) intrastriatally infused in a preclinical model of PD, obtained upon the administration of MPTP [30, 32]. After the unilateral transplantation into the striatum of MPTP-treated C57BL/6 mice, Er-NPCs were vital and capable of engrafting into the recipient’s brain. Er-NPC-treated animals improved their typical motor deficits within 3 days of cell transplantation, and this was accompanied by significant sparing of SN neurons. All these features and effects are likely dependent on erythropoietin’s (EPO) release since all of these were abolished by the co-injection of Er-NPCs with anti-EPO (aEPO) or anti-EPO-receptor (aEPO-R) antibodies. Little is known about the anti-inflammatory actions of Er-NPCs transplant in PD brains. Here, we focus on the study of this aspect and report that a rapid anti-inflammatory effect was evident 24 h after Er-NPCs transplant with the decrease of early pro-inflammatory cytokines mRNA levels (e.g., IL-1alpha, TNF). This effect was maintained for the following days, and IL-6 mRNA levels were significantly reduced as far as 7 days after transplantation. At the end of the 2-week observational period, histological data confirmed the reduction of activated microglia marker expression (GFAP and Iba1) and macrophages infiltration (CD68). Moreover, at the same time point, we observed the increase of markers associated with the M2-like protective phenotype. Co-injection of Er-NPCs with aEPO antibody neutralizes the Er-NPCs anti-inflammatory activity, strongly indicating that this effect is mediated by EPO released from Er-NPCs.

## Methods

### Animals and study approval

Procedures involving animals and their care were conducted in conformity with the Italian Guidelines for Laboratory Animals, which conform to the European Communities Directive of September 2010 (2010/63/UE), and the Review Committee of the University of Milan gave its approval to the study (No 2/2013). Male C57BL/6 mice (Charles River, Milan, Italy), 12–16 weeks old and weighing 20–24 g, were kept for at least 7 days before the experiments and housed in standard conditions (22 ± 2 °C, 65% humidity, and a 12-h light-dark cycle) with food and water ad libitum. Moreover, in order to make them amenable, the animals were accustomed to the behavioral tests (horizontal and vertical, see below for details) daily for 1 week prior to MPTP injection.

### Er-NPCs isolation

Erythropoietin-releasing neural precursors cells (Er-NPCs) expressing green fluorescent protein (GFP) were isolated 6 h postmortem from adult C57BL/6-Tg(UBC-GFP)30Scha/J mice weighing 25–30 g (Charles River) as previously described [29, 33, 34].

### Animal treatments

Experimental animals were divided into five groups: (1) control (CTRL, healthy animals, *n* = 24), (2) MPTP-treated mice (MPTP, *n* = 24), (3) MPTP-treated mice infused with PBS (SHAM, sham-operated; *n* = 18), (4) MPTP-treated mice transplanted with Er-NPCs (MPTP + Er-NPCs, *n* = 24), (5) MPTP-treated mice transplanted with Er-NPCs and anti-erythropoietin antibody (MPTP + Er-NPCs + aEPO, *n* = 12). Anti-erythropoietin antibody (sc-7956, Santa Cruz Biotechnology) was infused at the final concentration of 3 μg/ml [29, 30, 33, 35]. Parkinsonism was induced by intraperitoneal (i.p.) administration of 1-methyl-4-phenyl-1,2,3,6-tetrahydropyridine (MPTP) in C57BL/6 mice following the acute paradigm with a small modification. Briefly, animals were administered a double dose of MPTP hydrochloride: a first i.p. injection of MPTP (36 mg/kg) and a second i.p. injection of MPTP (20 mg/kg) after 7 days. Er-NPCs-treated animals were transplanted with 5 × 10^4^ cells/μl (5 μl) GFP expressing Er-NPCs, according to the following stereotaxic coordinates in relation to bregma: 0.1 mm posterior, 2.4 mm mediolateral, and 3.6 mm dorsal at the level of left striatum [36]. Please see Table 1 for a detailed number of animals for each group at specific time points. For gene expression analyses, animals from each group were sacrificed by cervical dislocation; their brains were removed and dissected. Immediately after dissection, the whole striatum, frontal cortex, and midbrain were frozen in dry ice until they were assayed. For immunohistochemistry analyses, animals from each group were anesthetized by i.p. injection of sodium pentobarbital (65 mg/kg), perfused through the left ventricle with 50 mL of saline solution, and fixed with 200 mL of 4% paraformaldehyde in 0.1 mol/L PBS. The brains were subsequently removed from the skulls and then cryoprotected at 4 °C in sucrose 300 g/L and 0.1 mol/L PBS solution for further sectioning.

**Table 1:** Number of animals used for experiments. All animals were tested for behavioral performances

Days post-transplant	Analysis	CTRL	MPTP	SHAM	MPTP + Er-NPCs	MPTP + Er-NPCs + aEPO
1	Real-time RT PCR	6	6	6	6	//
7	Real-time RT PCR	6	6	6	6	//
14	Real-time RT PCR	6	6	6	6	6
	Immunohistochemistry	6	6	//	6	6

// = no animals used for this analysis

### Behavioral tests

To investigate the recovery of motor dysfunction after cell transplantation, two different behavioral tests were performed: horizontal and vertical grid tests [30, 32, 37, 38]. Each animal was tested twice at each time point.

### Horizontal grid test

The grid apparatus was constructed according to Tillerson and co-workers [30, 32, 37]. The animal was videotaped for 30 s, and the videos were replayed for percentage forepaw fault analysis using a recorder with slow motion option. The number of unsuccessful forepaw steps divided by the total number of attempted forepaw steps was evaluated [37]. The mice were acclimatized to the grid twice a day for 1 week, before MPTP treatment. Three observers (ZG, FR, MM) in blind rated each trial for forepaw faults per step.

### Vertical grid test

The vertical grid apparatus was constructed according to Kim and co-workers [30, 32, 38]. For this test, the mouse was placed 3 cm from the top of the apparatus, facing upwards, and was videotaped when turning around and climbing down. The score reported was the time required by the mouse to make a turn, climb down, and reach the bottom of the grid with its forepaw within 180 s [30, 32, 38]. Before MPTP administration, mice were acclimatized to the grid twice a day for 1 week. The analysis was performed by three observers in blind (ZG, FR, MM).

### Olfactory test

For this test, mice were food deprived for 20 h before the test. A corn chip was buried under their bedding (1 cm) in a corner of the cage. Each mouse was positioned at the center of the testing cage, and the time to retrieve and bite the corn chip was measured [30]. The analysis was performed by three observers in blind (ZG, FR, MM).

### Immunohistochemistry and quantitative analysis

**Fig. 1:**
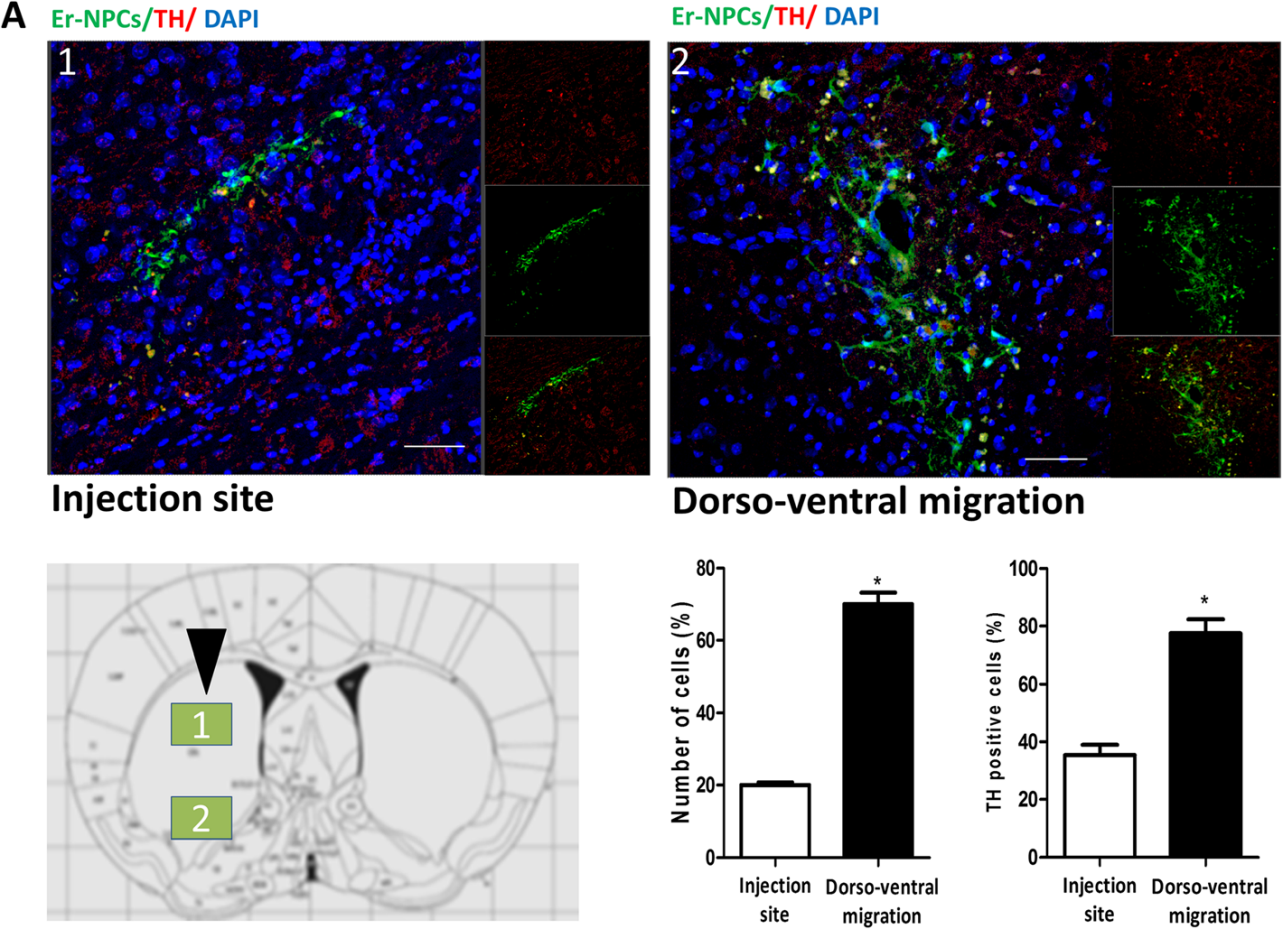
Er-NPCs engraft in the recipient’s brain and differentiate in TH-positive neurons. Confocal images of Er-NPCs intrastriatal distribution (green) 14 days after transplantation in the coronal sections taken from Er-NPCs-injected MPTP mouse brains (bars 50 μm). Co-expression of the cytoplasmic protein TH (red) and GFP (green) within a cell suggests dopaminergic differentiation of the transplanted Er-NPCs during migration. The histogram reports the quantification of positive cells to the TH marker in 18 different fields for each condition (*n* = 6 mice each group; 3 fields for mouse) (see the “Methods” section). Quantification was done by ImageJ picture analysis. *p < 0.05. Data reported in the histogram are expressed as average ± SD and refer to three brains for each experimental condition

Immunohistochemistry analyses were performed on 20 μm coronal section of the whole brain cut at − 25 °C using a cryostat (Leica), and slides were collected onto glass slides and rinsed with PBS and treated with blocking solution (10% NGS, 0.2% Triton X-100) following our previously published protocols [30, 32]. The following primary antibodies were used: monocyte/macrophages (MOMA/CD68, 1:25; Millipore), CD86 (1:200; Abcam), mannose receptor (CD206, 1:200; GeneTex), GFAP (1:1000; Covance), Iba1 (1:250; Abcam), CD11b (1:200; Abcam), tyrosine hydroxylase (TH, 1:500; Millipore), and DAT (1:200; Millipore). The following secondary antibodies were used: 546 goat anti-rabbit IgG (1:200; Alexa), 546 goat anti-mouse IgG (1:200; Alexa), 546 goat anti-rat IgG (1:200, Alexa), and 488 donkey anti-rabbit IgG (1:200, Alexa). Images were acquired using standardized confocal microscopy (Leica SP2 confocal microscope with He/Kr and Ar lasers; Heidelberg, Germany). Images of striatum immunostaining were acquired in the region corresponding to bregma 2.80/3.52 mm as indicated in the Paxinos and Franklin atlas [39]. Microphotographic digital analysis was performed using the ImageJ software [30, 32, 40]. The quantification of the positive pixels versus the negative background elicits an index score that includes the contributions from fibers and neuronal somata. The immunostaining conditions were the same for all sections analyzed; staining solutions and reaction times were the same. The microscope light intensity of the laser was the same for the analysis of all given brain sections and for determining the background optical density. Co-localization in Fig. 1 was analyzed over a 3D field obtained by stacking confocal planes acquired using standardized confocal microscopy (Leica SP (confocal microscope); Heidelberg, Germany).

### THP1 co-cultures with Er-NPCs

THP1 cells were seeded with fresh RPMI medium supplemented with 3% FBS on six trans-well plates at the concentration of 5 × 10^5^ cells/well for co-culture experiment and activated with 50 ng/ml PMA. Twenty-four hours after seeding, the THP1 was stimulated adding 1 μg/ml LPS for 1 h. For co-culture experiments, Er-NPCs were dissociated, counted, and re-suspended in the THP1 medium at the concentration of 4 × 10^5^ cells/well. Co-culture of THP1 and Er-NPCs with or without aEPO antibody (5 μg/ml) was performed using 0.4 μm pore size trans-well inserts (Corning). After 3 h of co-culturing, inserts were removed and the THP1 were isolated for RNA extraction and real-time RT-PCR. Dexamethasone was used (100 μM) as anti-inflammatory positive control [41].

### RNA extraction and real-time PCR

Gene expression analyses were performed 7 days after transplant (*n* = 6 mice for each group) in striatum homogenates [29, 33]. The left and right striatum regions were dissected out rapidly, frozen on dry ice, and stored at − 80 °C for further analyses. Total RNA was extracted using TRIZOL® reagent (Life Technologies) following the manufacturer’s instructions, quantified, and processed as described [42, 43]. Total RNA (1 μg) was reverse transcribed using iScript cDNA synthesis kit (Bio-Rad) according to the manufacturer’s instructions. Real-time RT-PCR was performed with StepOnePlus™ Real-Time RT-PCR System (Thermo Fisher) using iQ SYBR Green Supermix (Bio-Rad). Primers were designed using Oligo Perfect Designer Software (Life Technologies) and were checked using the BLASTn tool on NCBI (https://blast.ncbi.nlm.nih.gov/Blast.cgi?PAGE_TYPE=BlastSearch), assessing in silico the specific targeting of the gene. Furthermore, in each RT-PCR reaction, the Tm peak was analyzed, and only single peak results (indicating specific amplification of one product) were deemed valid. A negative control analysis with no cDNA template was also performed in each RT-PCR to ensure that no contamination was present when performing the reaction. Primer sequences are reported in Table 2. Amplification conditions were AmpliTaq activation 95 °C for 10 min, PCR denaturation step 95 °C for 15 s, PCR annealing and elongation step 60 °C for 1 min, and 40 cycles of PCR were performed. Genes were quantified in triplicates, and 18S was used as housekeeping gene. Gene expression was calculated using the 2^−ΔΔCt^ method.

**Table 2:** Primer sequences used to study gene expression

m 18S	F: AACTTTCGATGGTAGTCGCCGT
	R: TCCTTGGATGTGGTAGCCGTTT
m IL1-alpha	F: ATGGCCAAAGTTCCTGACTTGTTTGAAGAC
	R: GTTGCTTGACGTTGCTGATACTGTCACCCG
m IL1-beta	F: GGCAACTGTTCCTGAACTCAACTGTGAAAT
	R: CAGGTAGCTGCCACAGCTTCTCCACAGCCA
m IL-6	F: TCCAGTTGCCTTCTTGGGACTGATGCTGGT
	R: AGTTTCAGATTGTTTTCTGCAAGTGCATCA
m IL-10	F: CCTGGCTCAGCACTGCTATGCTGCCTGCTC
	R: AAGTAACCCTTAAAGTCCTGCATTAAGGAG
m TNF	F: GACGTGGAACTGGCAGAAGAGGCACTCCC
	R: GAGGCCATTTGGGAACTTCTCATCCCTTTG
h IL1-beta	F: ACAGATGAAGTGCTCCTTCCA
	R: GTCGGAGATTCGTAGCTGGAT
h TNF	F: CACTGAAAGCATGATCCGGGACGTGGAGCT
	R: TCTTCCCTCTGGGGGCCGATCACTCCAAAG
h 18S	F: GCTTAATTTGACTCAACACGGGA
	R: AGCTATCAATCTGTCAATCCTGTC

*m* mouse, *h* human

### Statistical analyses

Statistical analyses between groups were evaluated using GraphPadPrism 4.00 version, and data are expressed as mean ± SD. Behavioral data were analyzed with a two-way ANOVA model with time and group (CTRL, MPTP, SHAM, MPTP + Er-NPCs, and MPTP + Er-NPCs + aEPO) as factors. The null hypothesis was rejected when *p* < 0.05. Gene expression data were analyzed in triplicate and results were expressed as the average of six animals. The expression pattern of each gene was analyzed by one-way ANOVA followed by Bonferroni’s multiple comparisons test to assess statistical significance.

## Results

### Transplanted Er-NPCs integrate into the damaged host brain and promote a rapid recovery of function

**Fig. 2:**
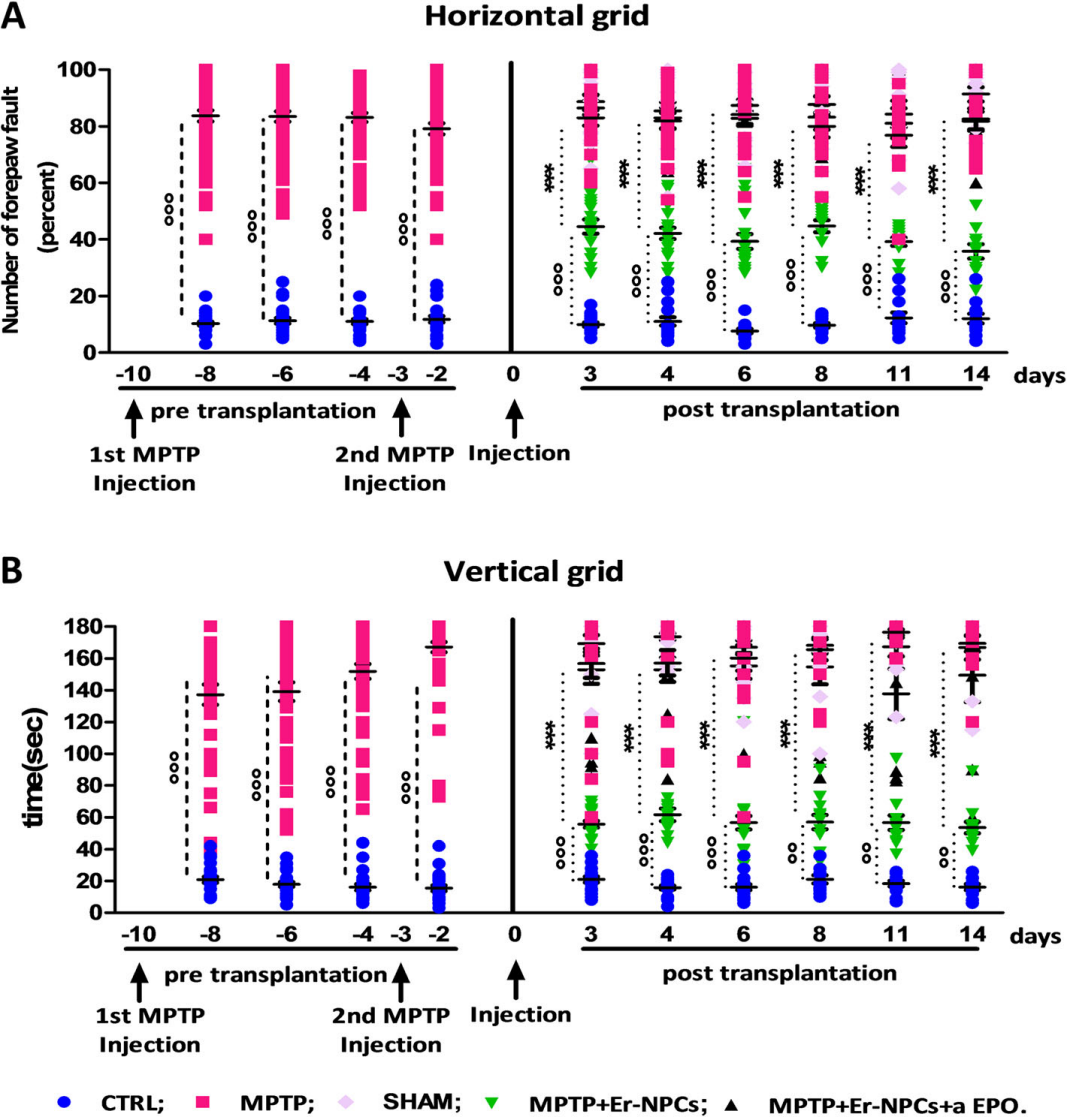
Er-NPCs promote functional recovery. Horizontal (**a**) and vertical (**b**) grid tests were used for the behavioral analysis, and the percentage of forepaw faults and the time for descending the grid are shown in the graphs. Five groups of animals were analyzed: (1) control (CTRL, healthy animals, *n* = 24), (2) MPTP-treated mice (MPTP, *n* = 24), (3) MPTP-treated mice infused with PBS (SHAM, sham-operated; *n* = 18), 4) MPTP-treated mice transplanted with Er-NPCs (MPTP + Er-NPCs, *n* = 24), and (5) MPTP-treated mice transplanted with Er-NPCs and anti-erythropoietin antibody (MPTP + Er-NPCs + aEPO, *n* = 12). Data are expressed as mean ± SD. Statistical analysis was performed with two-way ANOVA test followed by Bonferroni post-test. °°°*p* < 0.001; °°*p* < 0.01 vs CTRL; ***p < 0.001vs MPTP

The murine model of Parkinson’s disease was obtained by the administration of MPTP neurotoxin to C57BL/6 mice, following the paradigm reported previously [30, 32] (see the “Methods” section and Additional file 1 for details). Two weeks post-injection, transplanted Er-NPCs were engrafted in the recipient’s brain. Figure 1 shows that transplanted Er-NPCs migrate from the injection site to different brain sites. Their morphology looks more differentiated presenting a developed soma of good size with abundant neurite projections in the recipient striatum. Quantitative co-localization analyses performed in the striatum sections 2 weeks post-injection indicate that 70.01% (± 5.6) of engrafted Er-NPCs had migrated more than 3.25 mm following a dorso-ventral pathway, supporting previously reported results [30]. Migration is at least of 2.3 mm from the injection site following a rostro-caudal pathway (not shown) [30] and a minor percentage of cells (28.58% ± 1.42) even reached the substantia nigra ipsilateral and contralateral to the injection site [30]. A large portion of the grafted cells are localized within the striatum, more than 4–5 mm from the injection site, and express markers of mature neurons, such as Map2 and NeuN, while only a lower percentage of them was positive to NG2 and Nestin (82.30 ± 6.88% NeuN; 71.52 ± 9.45% MAP-2, 38.53 ± 5.81% NG2; 29.71 ± 12.20% Nestin) [30, 32]. TH expression is present in cells engrafted in the striatum and migrated far from the injection site (80% of GFP^+^-Er-NPCs localized more than 5 mm from the injection site) (Fig. 1). The evaluation of animals’ recovery of function was monitored with horizontal and vertical grid tests for 2 weeks after Er-NPCs infusion (2.5 × 10^5^ cells/brain) (Fig. 2) [30, 32]. The administration of the neurotoxin MPTP causes damages to the nigrostriatal projections. This leads to the increase of forepaw faults of the anterior paw, as it can be appreciated in the horizontal grid test (Fig. 2a), and of the time taken to descend from the grid in the specific test (Fig. 2b). The functional recovery due to intra-striatal injection of Er-NPCs is already detectable in the third day after the transplant as the percentage of forepaw faults in mice treated with the Er-NPCs is 44.55% ± 2.5% compared to 83.02 ± 2.76% of the MPTP animals group. The initial functional recovery observed in animals treated with Er-NPCs is maintained for the entire observational period. In the vertical grid test, the differences in score between motor features of treated mice (MPTP + Er-NPCs) and not treated mice (MPTP) are highly more appreciable (Fig. 2b). The action of Er-NPC transplantation on the promotion of functional performances is also appreciable on the recovery of olfactory capabilities caused by MPTP evaluated by means of olfactory test (Additional file 2) [30].

### Er-NPCs grafts promote the rescue of endogenous TH-positive projections in the recipient striatum

**Fig. 3:**
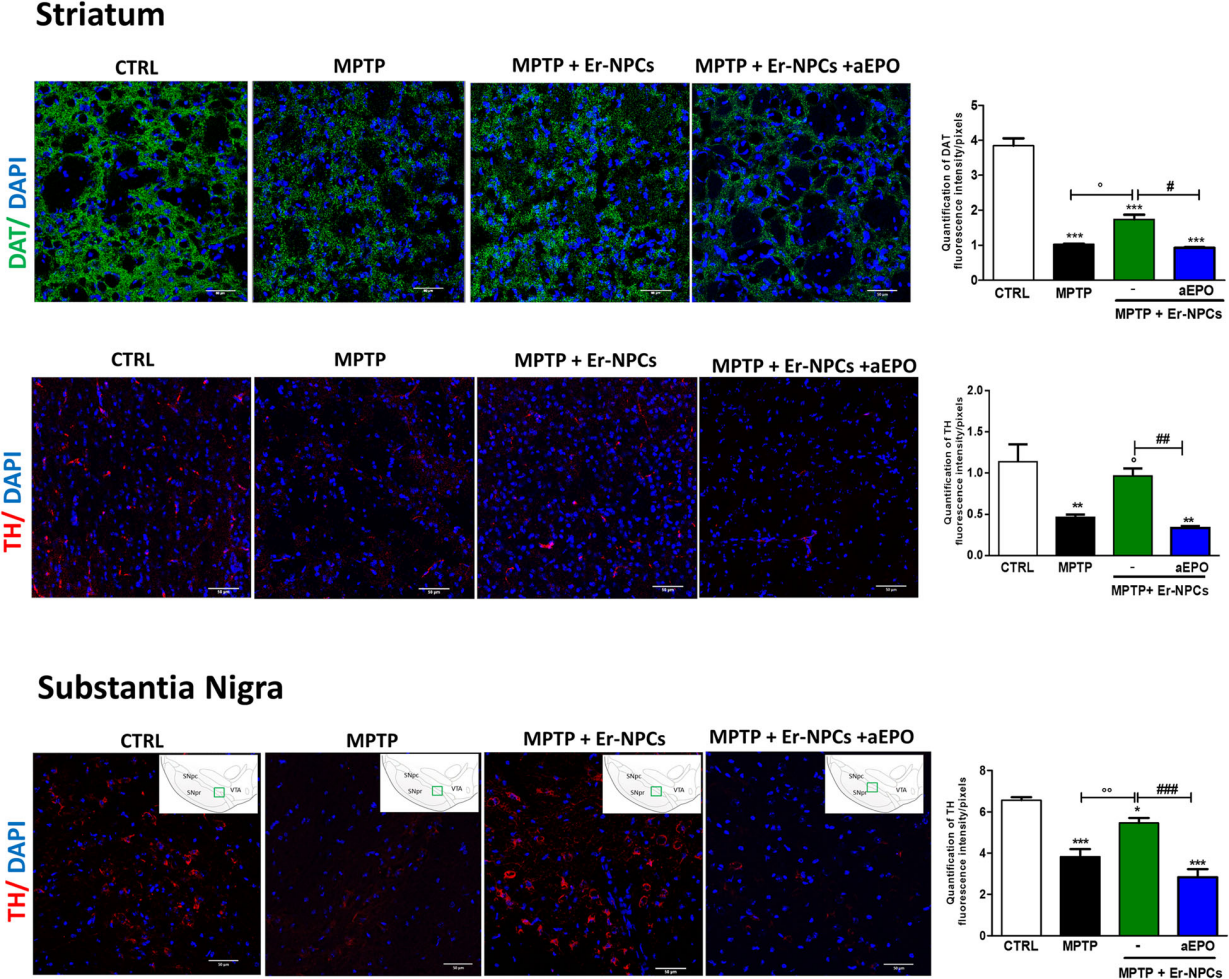
Er-NPCs promote the recovery of DAT and TH immunoreactivity. Confocal images of dopamine transporter (DAT) staining (green) (**a**) and tyrosine hydroxylase (TH) (showed in red) (**b**); of healthy mice (CTRL), mice treated with MPTP (MPTP), and MPTP infused with Er-NPCs (MPTP + Er-NPCs). Scale bars represent 50 μm. The quantification of fluorescence, represented in the diagram, was performed with the software ImageJ (NIH). The data reported in the diagram are referred to the mean ± SD (*n* = 6 mice each group; three fields for mouse). ***p < 0.001; *p < 0.05 vs CTRL; °°*p* < 0.01; °*p* < 0.05 vs MPTP; ^###^*p* < 0.001; ^#^*p* < 0.05 vs MPTP + Er-NPCs

The protective action of engrafted Er-NPCs was assessed by investigating the dopaminergic markers in the engrafted striatum. By performing quantitative confocal laser microscopy on striatal sections, we found that Er-NPCs significantly counteracted the MPTP-induced loss of both striatal DAT and TH-positive innervations (Fig. 3). The TH expression was also evaluated in the substantia nigra, and a concordant pattern was observed (Fig. 3) [30, 32].

### Er-NPCs transplantation counteracts pro-inflammatory cytokines expression in MPTP-recipient mice

**Fig. 4:**
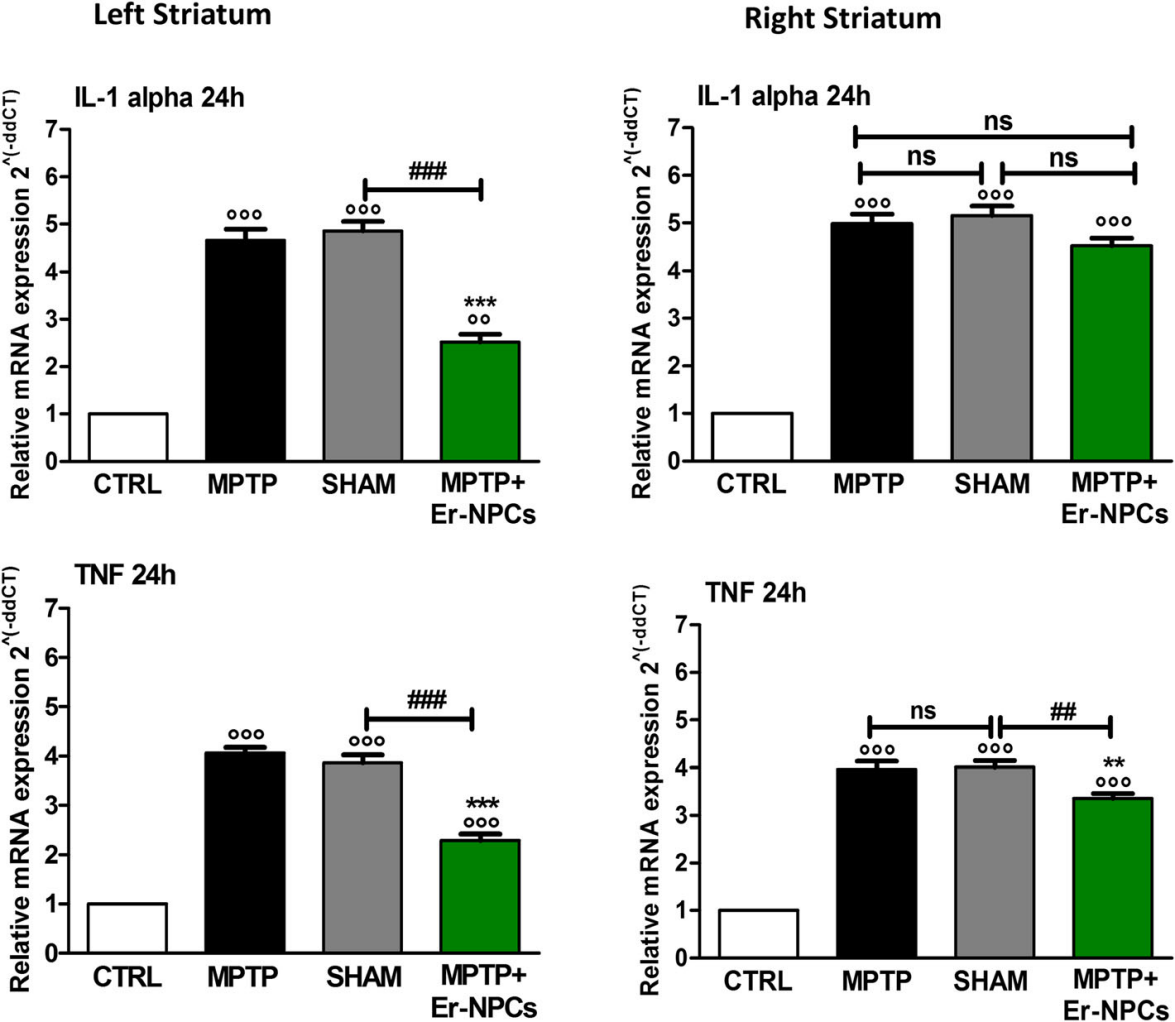
Evaluation of pro-inflammatory cytokines after 24 h (IL-1alpha, TNF). The levels of early cytokines were evaluated with real-time RT-PCR 24 h after Er-NPCs infusion (IL-1alpha, TNF). The quantification was conducted separately in the both hemispheres (right and left). 18S was used as housekeeping gene. This study was conducted in triplicate on six different brain samples. The data reported in the histogram are expressed as mean ± SD. °°°*p* < 0.001 vs CTRL; ***p < 0.001; **p < 0.01; *p < 0.05 vs MPTP; ^###^*p* < 0.001; ^##^*p* < 0.01 vs SHAM

**Fig. 5:**
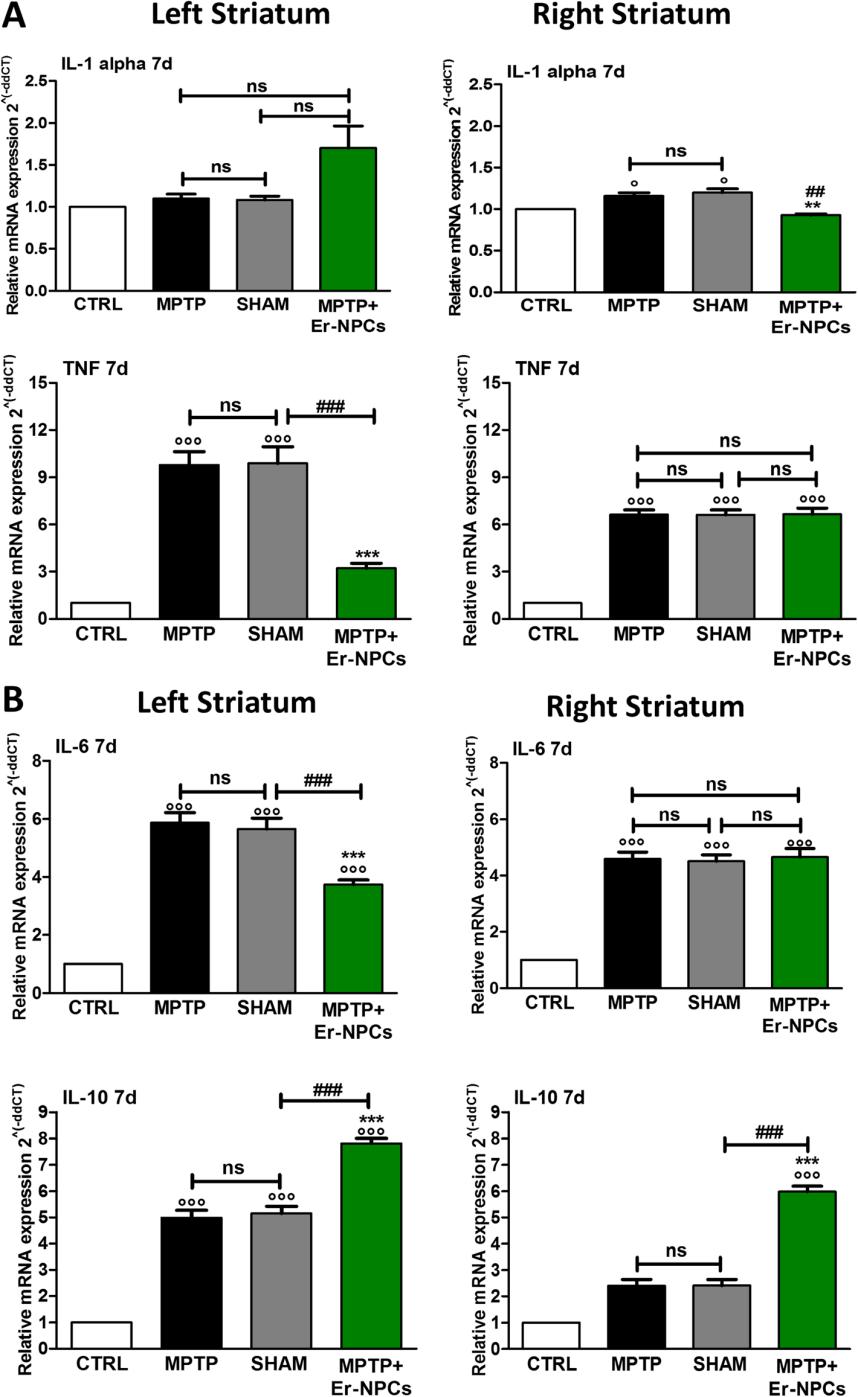
Evaluation of pro-inflammatory and anti-inflammatory cytokines (IL-1alpha, TNF; IL-6 and IL-10) 7 days after Er-NPCs transplant. The quantification was conducted by real-time RT-PCR separately in both hemispheres (right and left). 18S was used as a housekeeping gene. This study was conducted in triplicate on six different brain samples. The data reported in the histogram are expressed as mean ± SD. °°°*p* < 0.001 vs CTRL; ***p < 0.001; **p < 0.01; *p < 0.05 vs MPTP; ^###^*p* < 0.001; ^##^*p* < 0.01 vs SHAM

MPTP’s administration in models of PD is known to result in an inflammatory response mediated by an increased expression of neuro-inflammatory cytokines [2, 9, 44, 45]. We aimed to investigate the expression of neuroinflammatory cytokines, quantifying with real-time RT-PCR the mRNA levels of selected molecules. The MPTP administration causes a strong increase in the mRNA levels of the pro-inflammatory cytokines IL-1alpha and TNF (Fig. 4). The administration of Er-NPCs counteracts this phenomenon reducing these cytokines’ expression as early as 24 h after cells infusion (Fig. 4). This effect is significantly evident in the area of the striatum ipsilateral to the transplant. The expression of other interleukins (IL-6, IL-8, IL-10) and neurotrophic growth factors (BDNF, NGF) was also evaluated 24 h after Er-NPCs infusion without observing any significant differences between the Er-NPCs-treated group (MPTP + Er-NPCs) and the not-treated one (MPTP) (data not shown). The effect of the transplant is further maintained, as the significant reduction of TNF is still observed 7 days after the Er-NPCs infusion (Fig. 5a). IL-6, a pro-inflammatory cytokine, and IL-10, an anti-inflammatory interleukin, are highly expressed in the late response stage [45]. Seven days after the injection of Er-NPCs, IL-6 increased by sixfold in MPTP-treated mice compared to the correspondent cerebral area of control healthy animals (Fig. 5b). The levels of IL-6 were reduced in the left striatum (ipsilateral to the transplant) of Er-NPCs-infused MPTP mice, while the action of the transplanted cells was not evident in the right striatum of recipient mice (contralateral to the transplant). Conversely, the levels of expression of anti-inflammatory cytokine IL-10 were significantly increased in MPTP mice treated with Er-NPCs compared to the MPTP ones both in the left and right striatum (Fig. 5b).

### Er-NPCs regulate the expression of neuroinflammatory markers in the damaged striatum of recipient animals

**Fig. 6:**
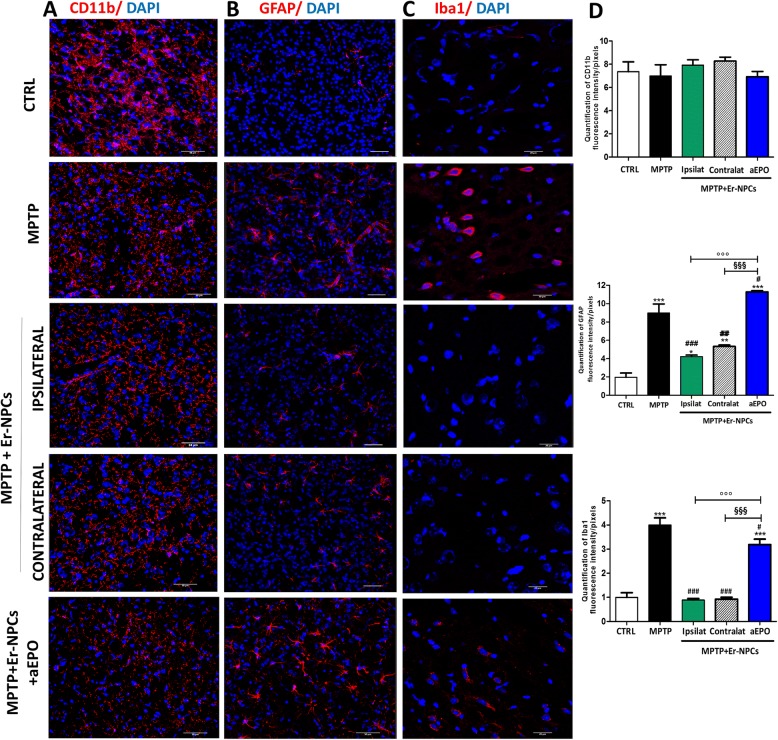
Er-NPCs counteract gliosis caused by MPTP. The coronal sections obtained from the brains of mice belonging to the following experimental groups (CTRL, MPTP, MPTP + Er-NPCs ipsilateral and contralateral, MPTP + Er-NPCs + aEPO ipsilateral) were labeled with the antibody CD11b (**a**), GFAP (**b**), and Iba1 (**c**) in red. Scale bars represent 50 μm (CD11b and GFAP) and 20 μm (Iba1). Quantification was done by ImageJ picture analysis software and reports the fluorescence intensity/pixel expressed by the studied markers in 18 different fields for each condition (*n* = 6 mice each group; 3 fields for mouse) (see the “Methods” section). ***p < 0.001; **p < 0.01; *p < 0.05 vs CTRL; ^###^*p* < 0.001; ^##^*p* < 0.01; ^#^*p* < 0.05 vs MPTP; °°°*p* < 0.001 vs MPTP + Er-NPCs ipsilateral; ^§§§^*p* < 0.001vs MPTP + Er-NPCs contralateral

Different studies demonstrated that stem cells have anti-inflammatory properties [26, 46], which lead us to think that the motor recovery we observe in animals treated with Er-NPCs could be mediated by Er-NPCs’ rapid counteraction of pro-inflammatory cytokines expression. Furthermore, we wished to clarify whether this is an effect mediated by EPO released by Er-NPCs during the engraftment in the recipient striatum [30, 32]. To dissect the action of Er-NPCs on counteracting neuroinflammatory processes, we set up a series of histological analyses performed at the end of observational period (2 weeks after Er-NPCs infusion) in which a group of Er-NPC-treated animals were also co-infused with anti-erythropoietin antibody. Figure 6a shows that there are no significant changes in CD11b’s expression, a well-characterized pan-macrophage and microglia marker [47, 48], in MPTP mice or after Er-NPCs or Er-NPCs + aEPO administration. Quantitative data are reported in the histogram that shows the quantification of fluorescence intensity (Fig. 6d) and the number of cells positive to CD11b marker expression (Additional file 3). To evaluate the presence of activated astrocytes, the levels of glial fibrillary acidic protein (GFAP) were investigated (Fig. 6b) [29]. GFAP resulted increased in the striatum slides of MPTP and MPTP + ErNPCs + aEPO-treated mice, while it decreased in Er-NPCs-treated mice, both in the ipsilateral and in the contralateral striatum. The expression levels were quantified for fluorescence intensity and reported in the histogram of Fig. 6d. To investigate the presence of immune cells, we studied the microglia in the striatum after the inflammatory stimulus, evaluating the expression of Iba1 (ionized calcium-binding adapter molecule 1), a protein codified by the AIF4 gene, which indicates the presence of activated macrophages in inflamed tissues [49]. Figure 6c shows that in the striatum of MPTP animals, there is an overexpression of the Iba1 protein. In the group of MPTP mice transplanted with Er-NPCs, the presence of Iba1 resulted significantly reduced and comparable to the control healthy animals both in the left and right striatum (Fig. 6c). Furthermore, in the striatal sections obtained from MPTP animals treated with Er-NPCs + aEPO, the downregulation of Iba1 expression was prevented. Indeed, fluorescence’s quantification shows that in this case, the signal is comparable to that of sections obtained from animals belonging to the MPTP group (Fig. 6d). These data are confirmed also by the stereological counts of cells positive to the investigated markers (Additional file 3).

**Fig. 7:**
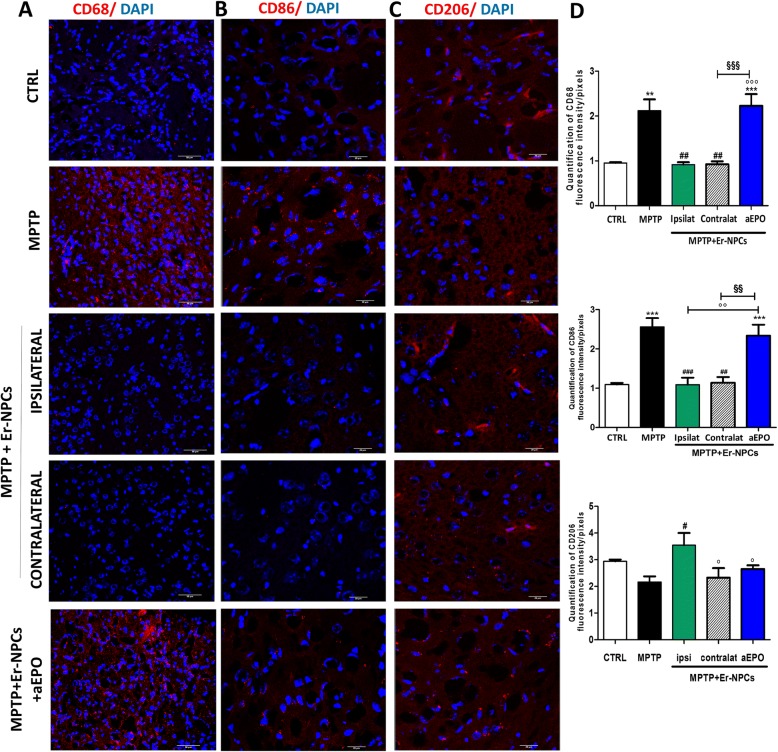
Er-NPCs engraftment counteracts macrophages invasion and allows the switch from M1 to M2 subtype. Immunohistochemistry analysis of coronal sections obtained from the brain of healthy control animals, treated with MPTP and with MPTP + Er-NPCs ipsilateral and contralateral to the transplant site and with MPTP + Er-NPCs + aEPO ipsilateral to the transplant site. The brain sections were labeled with the antibody CD68 (**a**), CD86 (**b**), and CD206 (**c**) in red. Scale bars represent 50 μm (CD68, CD86, and CD 206). Quantification was done by ImageJ picture analysis software and reports the fluorescence intensity/pixel expressed by the studied markers (*n* = 6 mice each group; three fields for mouse) (see the “Methods” section). ***p < 0.001; **p < 0.01 vs CTRL; ^###^*p* < 0.001; ^##^*p* < 0.01; ^#^*p* < 0.05 vs MPTP; °°°*p* < 0.001; °°*p* < 0.01; °*p* < 0.05 vs MPTP + Er-NPCs ipsilateral; ^§§§^*p* < 0.001; ^§§^*p* < 0.01 vs MPTP + Er-NPCs contralateral (**d**)

Studies in acute MPTP mouse models provided evidence of monocyte’s infiltration in the brain [50–52]. To investigate the levels of macrophages infiltration, we evaluated the expression of CD68, a marker associated with the lysosomal activity of myeloid cells [48, 53]. CD68 increases after the administration of MPTP neurotoxin (Fig. 7a), and in the ipsilateral and contralateral sections of the striatum transplanted with Er-NPCs, its immunoreactivity is significantly decreased. The histograms shown in panel d of the same figure report the quantification of fluorescence (Fig. 7d). The microglia has two different activation phenotypes, defined as M1-like (with a pro-inflammatory action) and M2-like (with an anti-inflammatory action) [54]. These two phenotypes develop different actions depending on their released mediators. The M1-like phenotype releases pro-inflammatory cytokines and other pro-inflammatory molecules such as NO and ROS. On the contrary, M2-like microglia release anti-inflammatory cytokines [20]. To discriminate between the two macrophages subtypes that infiltrate the degenerated striatum (M1 vs M2), we studied the immunoreactivity to phenotype-specific markers. In particular, we investigated the expression of CD86, marker of macrophages M1-like (Fig. 7b) and of CD206, a mannose receptor, and a typical marker of M2-like macrophages (Fig. 7c) [20]. The expression of CD86 was significantly increased in MPTP mice, while in mice treated with Er-NPCs, the levels of CD86 decreased, both in the ipsi and in the contralateral sites to the transplant (Fig. 7b). In MPTP mice co-administrated with Er-NPCs and aEPO antibody, the reduction of CD86 mediated by the cells was inhibited. An opposite pattern was observed for the CD206 marker, with a very low expression of this marker in MPTP mice and MPTP mice treated with Er-NPCs + aEPO (Fig. 7c). CD206 resulted in a high increase in MPTP mice treated with Er-NPCs (Fig. 7c). The quantification of fluorescence intensity relative to the expression of each marker is reported in Fig. 7d. Moreover, these observations are confirmed also by the stereological counts of cells positive to the investigated markers (Additional file 4).

### The anti-inflammatory effect of Er-NPCs is mediated by EPO

**Fig. 8:**
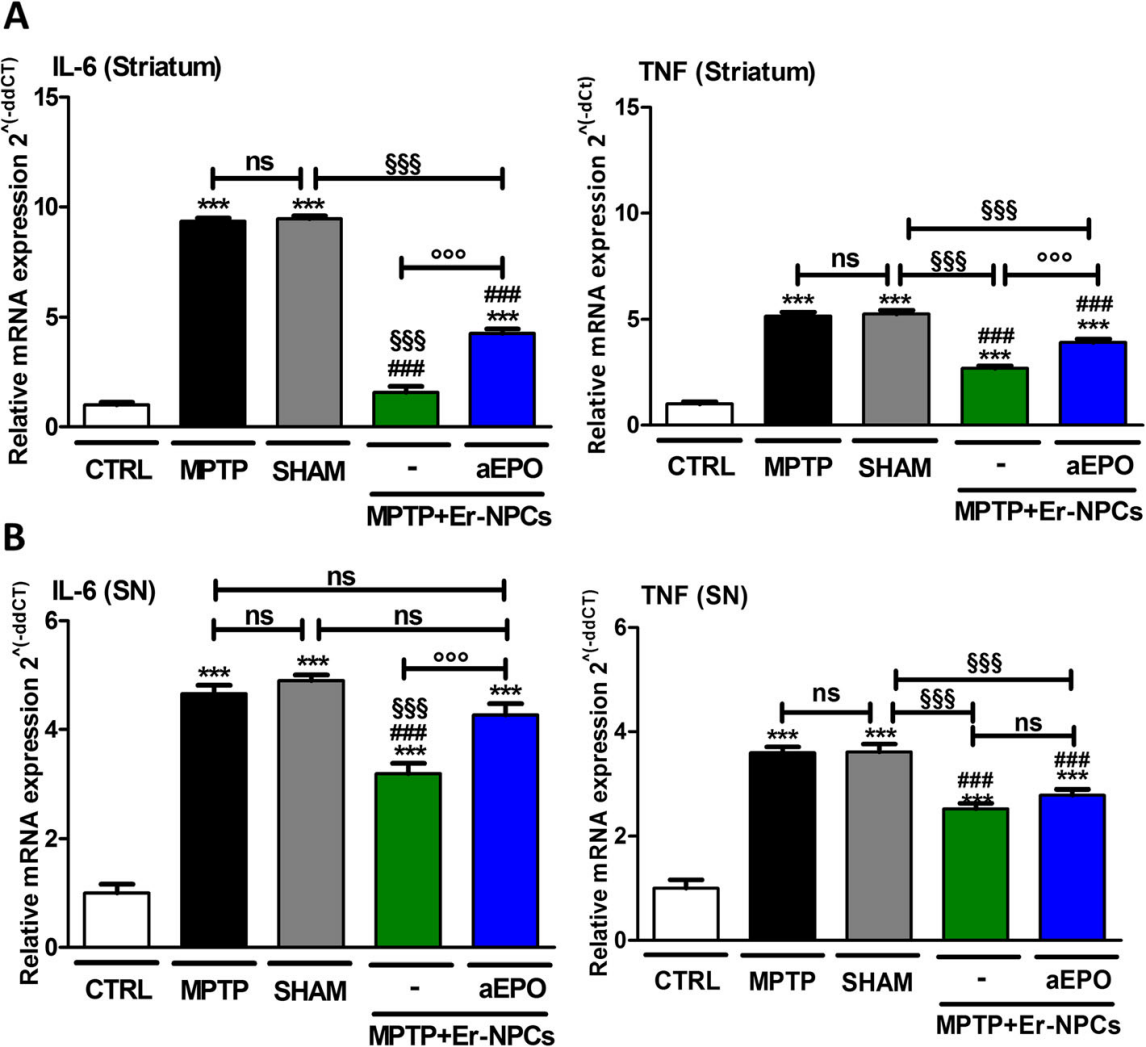
The co-administration of aEPO with Er-NPCs abrogated their positive effect on pro-inflammatory cytokines. At 2 weeks after transplantation, mRNA levels of IL-6 and TNF were quantified in the striatum (**a**) and substantia nigra (**b**). 18S was used as a housekeeping gene. The evaluation was performed in triplicate on six different brain samples. The data reported in the histogram are expressed as mean ± SD. ***p < 0.001 vs CTRL; ^###^*p* < 0.001 vs MPTP; ^$$$^*p* < 0.001 vs SHAM; °°°*p* < 0.001 vs MPTP + Er-NPCs

To confirm that the anti-inflammatory actions exerted by the Er-NPCs transplanted were connected to the release of EPO, at the end of the observational period (2 weeks after transplantation) six brains for each experimental group were explanted without perfusion. The striatum and substantia nigra were dissected from each brain, total RNA was extracted, and the RNA expression of pro-inflammatory cytokines IL-6 and TNF was evaluated (Fig. 8). The results of the expression analysis show that Er-NPCs transplant significantly allows the reduction of pro-inflammatory cytokines in both the striatum and substantia nigra of recipient animals (Fig. 8). The co-administration of Er-NPCs with aEPO antibody inhibits the therapeutic effect of transplanted cells. Indeed, in these animals and in the MPTP non-transplanted animals, the levels of all the studied cytokines resulted in significant increase as opposed to control. This observation is in complete concordance with what has been observed by immunofluorescence analysis of neuro-inflammatory markers.

### Er-NPCs override the pro-inflammatory cytokine production by macrophages in vitro

**Fig. 9:**
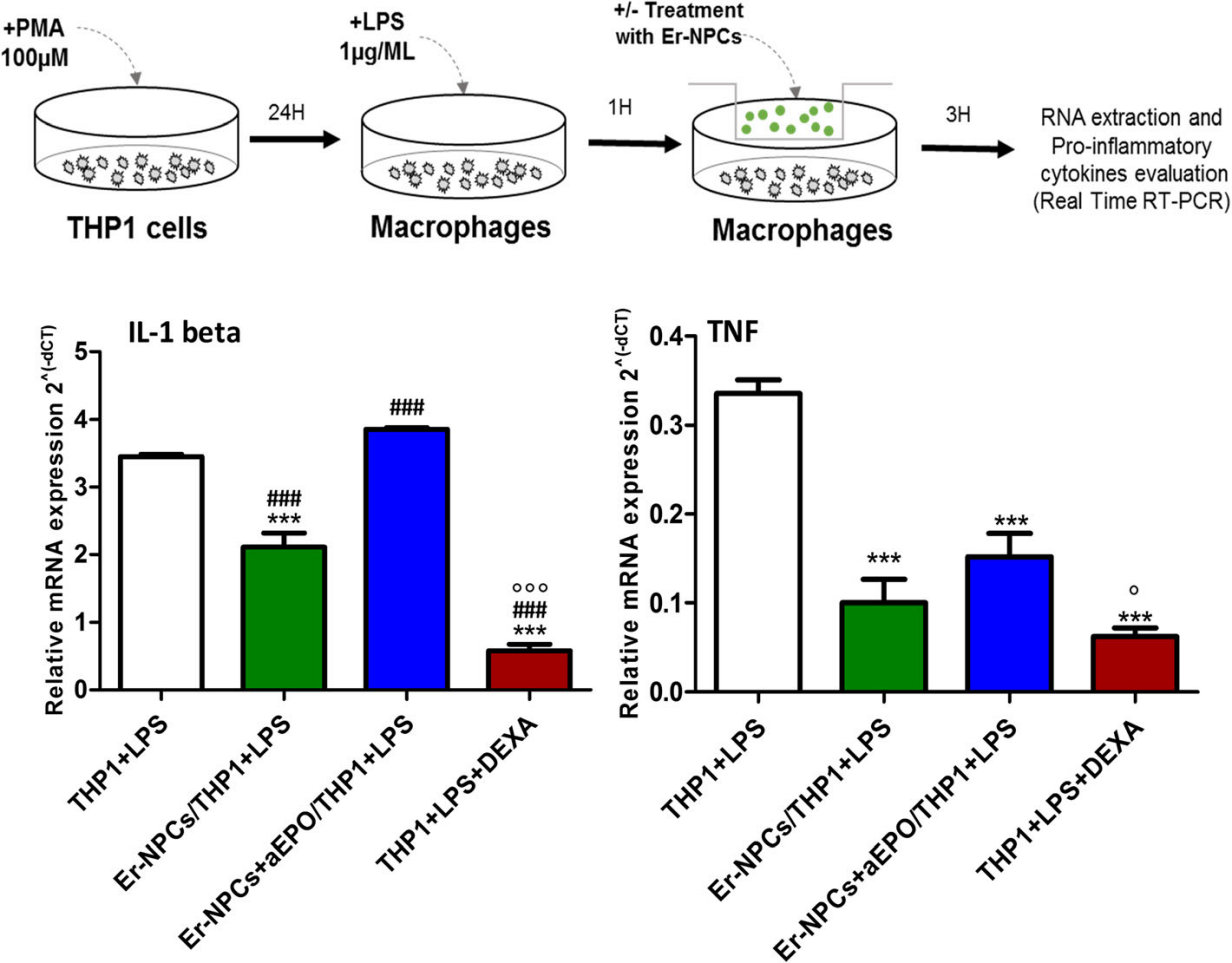
Er-NPCs inhibit the expression of pro-inflammatory cytokines in co-culture experiment. **a** Schematic of experimental set up for in vitro THP1 cells activation to macrophages and co-cultures with Er-NPCs. The assay was performed in trans-well. **b** mRNA expression levels of pro-inflammatory cytokines IL-1beta and TNF in co-culture experiments. mRNA levels were evaluated by real-time RT-PCR in not-treated activated THP1 versus co-culture with Er-NPCs, Er-NPCs + aEPO, and activated THP1 treated with dexamethasone (100 μM) a potent anti-inflammatory and immunosuppressive agent used as control. The co-culture assay lasted for 3 h. The experiment was repeated trice, and each point was assayed in triplicate. The data reported in the histogram are expressed as mean ± SD. ***p < 0.001 vs THP1 + LPS; ^###^*p* < 0.001 vs Er-NPCs/THP1 + LPS; °°°*p* < 0.001; °*p* < 0.05 vs Er-NPCs + aEPO/THP1 + LPS

To further investigate whether the anti-inflammatory properties were due to the transplanted neural precursors, we performed an expression analysis of pro-inflammatory cytokines IL1-beta and TNF using an in vitro system that recapitulates the inflammatory microenvironment. THP1 cells were differentiated with PMA for 24 h (100 μM) and activated using LPS for 1 h (1 μg/ml) [55] (please see schematic representation in Fig. 9). Then, Er-NPCs were added using a trans-well approach and maintained in the co-culture trans-well system for 3 h. As expected, the THP1 macrophages showed a significant increase in the expression levels of TNF and IL1-beta mRNAs. The presence of Er-NPCs counteracted the expression of both pro-inflammatory cytokines with respect to THP1 macrophages (Fig. 9). The co-incubation with Er-NPCs in presence of anti-EPO antibody (5 μg/ml) partially inhibited the effect exerted by Er-NPCs (Fig. 9).

## Discussion

Many reports have shown the ability of transplanted neural stem cells to modify the environment of the recipient tissue and give rise to positive effects in animal experimental models of PD. Here, we uncover the anti-inflammatory action exerted by engrafted Er-NPCs in an MPTP model of PD. Their transplantation promoted functional recovery and resulted in the sparing of dopaminergic nigro-striatal projections. By perceiving signals coming from the microenvironment of damaged tissue, stem cells can migrate to specific sites in the body and respond to a specific signal by releasing cytokines and modifying their own fate [56]. All of these features allow stem cells to be a possible therapeutic approach for many neurodegenerative diseases, including PD.

Here, we used accessible, stably expandable, and well-characterized erythropoietin-releasing neural precursors cells (Er-NPCs) [29–34]. The aim of this work was to investigate the mechanisms exerted by Er-NPCs transplantation in a mouse model of PD, obtained with MPTP administration, which mimics the inflammatory process related to the pathology [50–52, 57].

Transplanted Er-NPCs survived after transplantation, differentiated in the recipient striatum, and induced significant amelioration in functional motor symptoms caused by MPTP intoxication 3 days post-transplantation, as well as reduced pro-inflammatory cytokines 24 h after the infusion. These results support the hypothesis that the transplanted Er-NPCs inhibit the neuroinflammatory phenomena associated with Parkinsonism induced by the administration of MPTP neurotoxin, via an EPO-dependent mechanism. Moreover, although the expression of specific markers of macrophages polarization is not completely categorized [58], the transplanted neural precursors interact with the recipient microenvironment and stimulate tissue response to inflammation, supporting the transition of macrophages from M1-like phenotype to M2-like, towards a more protective action [59]. It would require specific assays such as RNASq experiments to dissect deeply the macrophages pathways involved. We have observed that there is no direct contact between M2-like macrophages (positive to CD206) and transplanted Er-NPCs, suggesting that the observed transition does not require the direct contact with the cells but can probably be ascribed to a paracrine phenomenon. The mechanism could be related to a local counteraction of inflammation and neuroprotective action mediated by EPO [30, 32, 59–61] on neurons and neural processes affected by MPTP. Neuroinflammation is a physiologic response to the administration of the MPTP toxin resulting in microglia activation which secretes multi-functional immunoregulatory factors, most notably TNF, IL-1 and IL-6 families, interferon gamma (IFN-gamma), and transforming growth factor beta (TGF-beta), [62, 63]. All of these factors act in context-dependent ways to modulate inflammatory processes and the permeability of the BBB [15, 64, 65]. Anti-inflammatory properties of Er-NPCs are shown by the fact that their transplant downregulates IL-6 and TNF mRNA levels and reduces macrophages invasion in the striatum and in the SNpc of recipient mice.

This is consistent with our previous work performed in experimental traumatic spinal cord injury, which reported the reduction of inflammatory cytokine levels and macrophage infiltration after the treatment with rhEPO [60] or administration of Er-NPCs [29, 33]. This seems to happen also in the present work, as there is a counter-action of the inflammatory events caused by MPTP, particularly ipsilateral to the injection site. Moreover, we observed here that transplanted Er-NPCs were able to counteract the expression of activated microglia’s markers both in the ipsi- and contra-lateral striatum. This further validates our previous evidences [30, 32] showing that the inhibition of EPO’s release by Er-NPCs influenced the striatum microenvironment by reducing endogenous EPO expression [32]. Thus, we could speculate that the action on contralateral neuroinflammation can be ascribed both to the diffusion of EPO released by engrafted precursors and by the modification of the local microenvironment [32]. The counteractive effects on astrocytes (GFAP staining reduction) are quite relevant since this type of cells are highly activated in PD and produce an elevated level of inflammatory cytokines and reactive oxygen species [66]. Moreover, such an activation is correlated with increased neuronal death [67, 68].

We found that the levels of pro-inflammatory cytokines, such as IL-1alpha, TNF, and IL-6 mRNA levels, are significantly reduced 24 h (IL1-alpha, TNF) and 1 week (IL-6) after Er-NPCs administration in contrast with IL-10, an anti-inflammatory cytokine, which resulted increased in the ipsilateral striatum 1 week after administration of Er-NPCs. The variation in the mRNA levels of cytokines is concordant with the decrease of CD86 (marker of macrophage phenotypes M1-like) and the increase of the level of CD 206 (marker of macrophages M2-like). Moreover, our results suggest that EPO released by Er-NPCs is implicated in the shift from a pro-inflammatory to a positive phagocytic state [58, 59, 69]. Phenotypic M changes are associated with no major changes in overall CD11b immunoreactivity in MPTP-treated animals transplanted with Er-NPCs with respect to CTRL. This could be explained by the fact that both resident microglia and infiltrated macrophages are positive to CD11b staining following central nervous system injuries [47, 48]. It has been shown that both phenotypes have phagocytic functions: under pro-inflammatory conditions M1-like macrophages, being toxic cells, target viable neurons, cause their death, and help in the propagation of the damage. On the other hand, under anti-inflammatory stimuli, M2-like subtype macrophages, being protective, with their phagocytic activity remove toxic cellular debris and dying cells to allow the creation a favorable microenvironment for the recovery [70, 71]. The ability of Er-NPCs to influence and limit the “pro-inflammatory activity” might be a key pathway to confer protection from PD.

This work confirmed that after the infusion, most of Er-NPCs survive (≥ 75%) [32] in the striatum and differentiate mainly in neurons [32]. The engrafted precursors migrate in the distal zones from the injection site and show a higher capacity to differentiate: 60% of these cells are positive to MAP2, and more than 80% express the NeuN marker.

This data, if considered together with the results obtained from the transplant of Er-NPCs in traumatic spinal cord injury model [29, 30], shows that these neural precursors are able to interact with the microenvironment where they are infused and differentiate to specific lineages [29, 32].

One of the most encouraging results of the pre-clinic approach that characterizes this work is the study of functional recovery in animals treated with Er-NPCs. Indeed, the motor recovery is already evident at the third day after transplant; it is further improved during the full observational period of 2 weeks, and it is maintained up to 2 months after the transplant [30]. The positive results obtained from the analysis of the motor capabilities are supported by observations obtained via immunohistochemistry analysis that demonstrate that precursor’s transplantation promotes the recovery of TH projections and DAT expression in the recipient striatum associated with the recovery of TH-positive cellular bodies in the SNpc [30]. Aiming to characterize the mechanism of action of injected Er-NPCs, we have studied the role of EPO when released by Er-NPCs [34] in vivo and in vitro. The inhibition of EPO’s action, caused by the co-administration of the aEPO antibody both in the in vivo and in the in vitro macrophage activation assay, allowed us to validate that the release of this cytokine by the Er-NPCs is one of the mechanisms responsible for their capability to counteract neuroinflammation. EPO’s neuroprotective and anti-inflammatory effects are very well studied and widely supported in the literature [72]. EPO analogs or non-erythropoietic-mutant EPO variants showed neuroprotective actions in MPTP-induced neurotoxicity and had neuro-rescue effects in rodent models of PD [73–76]. Interestingly, the administration of aEPO antibody in the in vitro macrophages activation assay only partially suppresses the effect of Er-NPCs on TNF mRNA expression levels. This suggests that the feature of Er-NPCs to act as biological disease-modifying agents should not only be due to EPO but possibly to other unknown anti-inflammatory molecules released by these cells.

## Conclusion

In conclusion, the data presented in this work suggests that the adult neural precursors releasing EPO represent an interesting model of stem cell therapy approach. Er-NPCs are able to interact with and improve the microenvironment of the damaged tissue thanks to the release of protective factors such as EPO. Moreover, our data suggests that erythropoietin can be a potential pharmacological therapy useful for the treatment of Parkinson’s disease, thanks to its anti-inflammatory properties.

## Additional files

Additional file 1: Experimental plan. (PDF 214 kb)

Additional file 2: Er-NPC-treated animals recover olfactory capabilities. Five groups of animals were analyzed with this test: 1) Control (CTRL, healthy animals, *n* = 24); 2) MPTP treated mice (MPTP, *n* = 24); 3) MPTP treated mice infused with PBS (SHAM, sham operated; *n* = 18); 4) MPTP treated mice transplanted with Er-NPCs (MPTP + Er-NPCs, n = 24) 5) MPTP treated mice transplanted with Er-NPCs and anti-erythropoietin antibody (MPTP + Er-NPCs + aEPO, *n* = 12). Data are expressed as mean ± SD. Statistical analysis was performed with two-way ANOVA test followed by Bonferroni post-test. °*p* < 0.05 vs CTRL; ***p < 0.001 vs MPTP. (PDF 197 kb)

Additional file 3: Percentage of cells positive to CD11b, GFAP, and Iba1. Graphs report the stereologic counts of positive cells to investigated markers quantified in nine different fields for each condition (three mice for each group). The analysis was performed 2 weeks after Er-NPCs injection. Quantification was done by ImageJ picture analysis software. Data are expressed as mean ± SD. ***p < 0.001; **p < 0.01 vs CTRL; ^###^*p* < 0.001; ^##^*p* < 0.01 vs MPTP; °°°*p* < 0.001; °*p* < 0.05 vs MPTP + Er-NPCs ipsilateral; ^§§§^*p* < 0.001 vs MPTP + Er-NPCs contralateral. (PDF 185 kb)

Additional file 4: Percentage of cells positive to CD68, CD86, and CD206. Graphs report the stereological counts of positive cells to the investigated markers quantified in nine different fields for each condition (three mice for each group). The analyses were performed by evaluating nine different fields for each condition (three mice for each group). Quantification was done by ImageJ picture analysis software. Data are expressed as mean ± SD. ***p < 0.001; **p < 0.01; *p < 0.05 vs CTRL; ^###^*p* < 0.001; ^#^*p* < 0.05 vs MPTP; °°°p < 0.001 vs MPTP + Er-NPCs ipsilateral; ^§§§^*p* < 0.001; ^§^*p* < 0.05 vs MPTP + Er-NPCs contralateral. (PDF 181 kb)

## Abbreviations

aEPO: Anti-EPO
aEPO-R: Anti-EPO-R
ANOVA: Analysis of variance
BBB: Blood-brain barrier
BDNF: Brain-derived neurotrophic factor
CNS: Central nervous system
CTRL: Control
DA: Dopaminergic
DAT: Dopamine transporters
DEXA: Dexamethasone
EPO: Erythropoietin
EPO-R: Erythropoietin receptor
Er-NPCs: Erythropoietin releasing neural precursors cells
FBS: Fetal bovine serum
GFP: Green fluorescent protein
IFN: Interferon
IL: Interleukin
LPS: Lipopolysaccharide
MOMA: Monocyte/macrophages
MPTP: 1-Methyl-4-phenyl-1,2,3,6-tetrahydropyridine
NGF: Nerve growth factor
NGS: Normal goat serum
PBS: Phosphate-buffered saline
PD: Parkinson’s disease
PMA: Phorbol 12-myristate 13-acetate
RPMI: Roswell Park Memorial Institute medium
SNpc: Substantia nigra pars compacta
TH: Tyrosine hydroxylase
THP1: Human leukemia monocytic cell line
TNF: Tumor necrosis factor
