# Supplementary figures and images for: Counteracting neuroinflammation in experimental Parkinson’s disease favors recovery of function: effects of Er-NPCs administration
Source: J Neuroinflammation. 2018 Nov 30;15:333. doi: 10.1186/s12974-018-1375-2 (PMC6271641; doi:10.1186/s12974-018-1375-2)

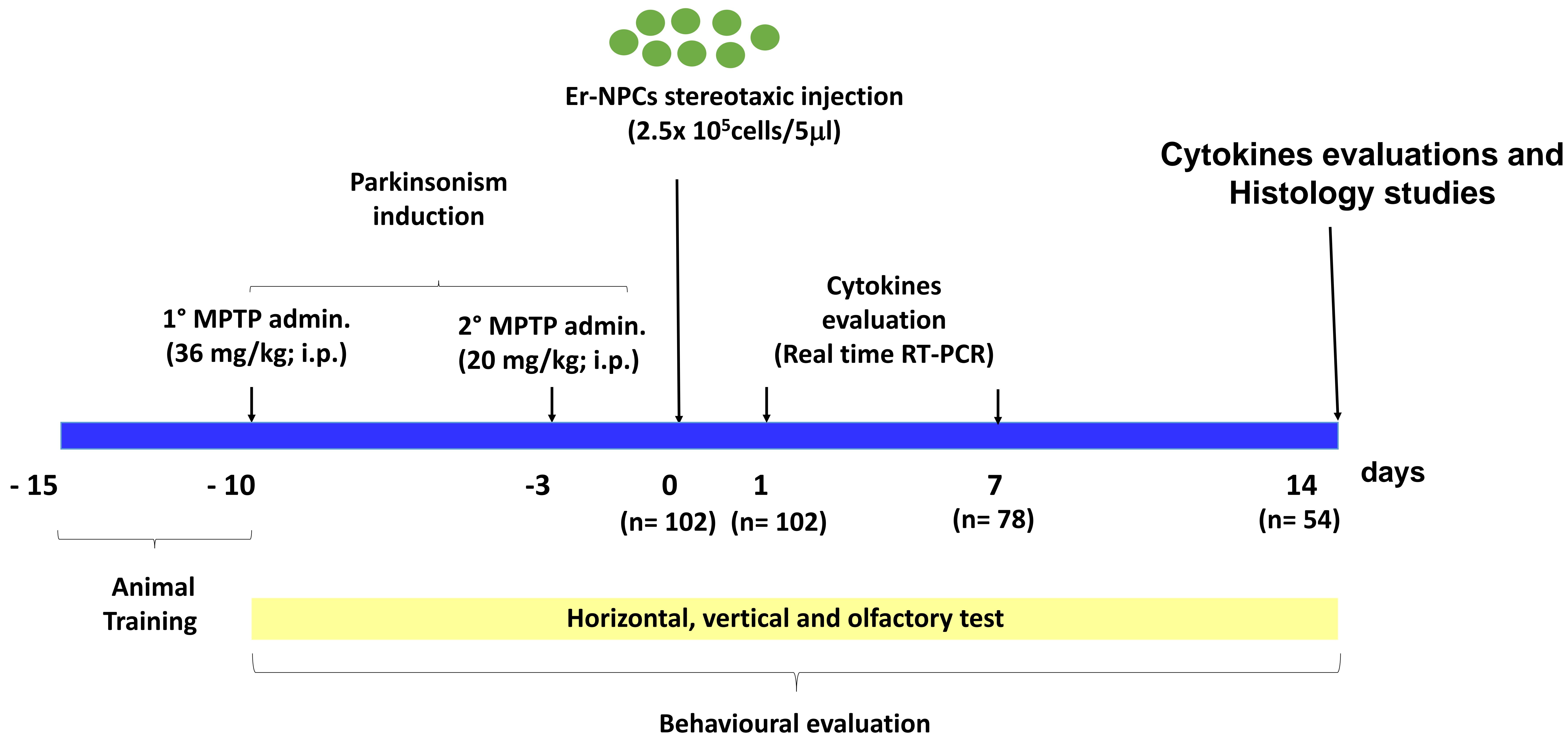

Supplement: Supplementary file 1 — Experimental plan. (PDF 214 kb) [file 12974_2018_1375_MOESM1_ESM.pdf]

# Olfactory Test

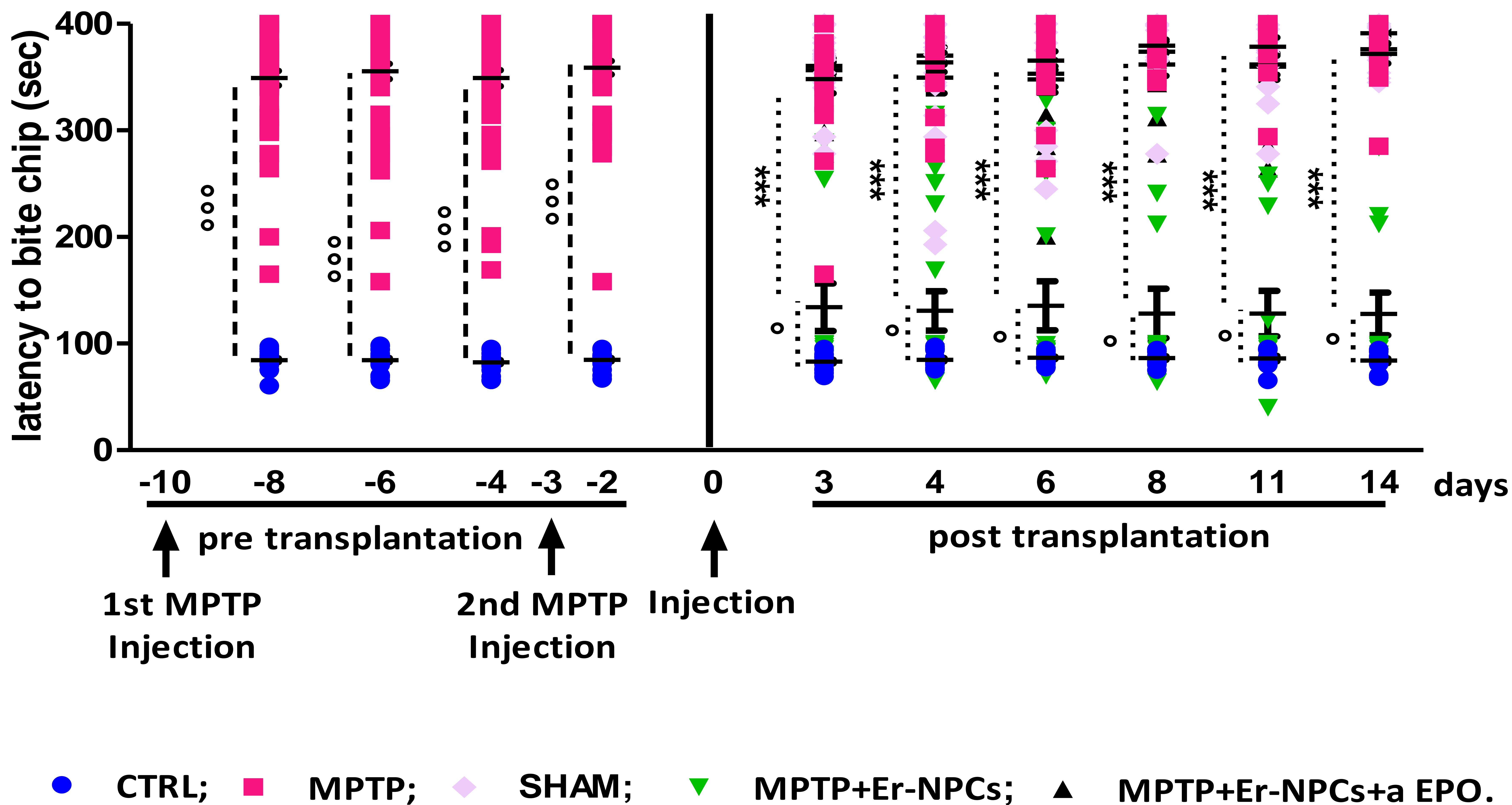

Supplement: Supplementary file 2 — Er-NPC-treated animals recover olfactory capabilities. Five groups of animals were analyzed with this test: 1) Control (CTRL, healthy animals, n = 24); 2) MPTP treated mice (MPTP, n = 24); 3) MPTP treated mice infused with PBS (SHAM, sham operated; n = 18); 4) MPTP treated mice transplanted with Er-NPCs (MPTP + Er-NPCs, n = 24) 5) MPTP treated mice transplanted with Er-NPCs and anti-erythropoietin antibody (MPTP + Er-NPCs + aEPO, n = 12). Data are expressed as mean ± SD. Statistical analysis was performed with two-way ANOVA test followed by Bonferroni post-test. °p < 0.05 vs CTRL; ***p < 0.001 vs MPTP. (PDF 197 kb) [file 12974_2018_1375_MOESM2_ESM.pdf]

**A**

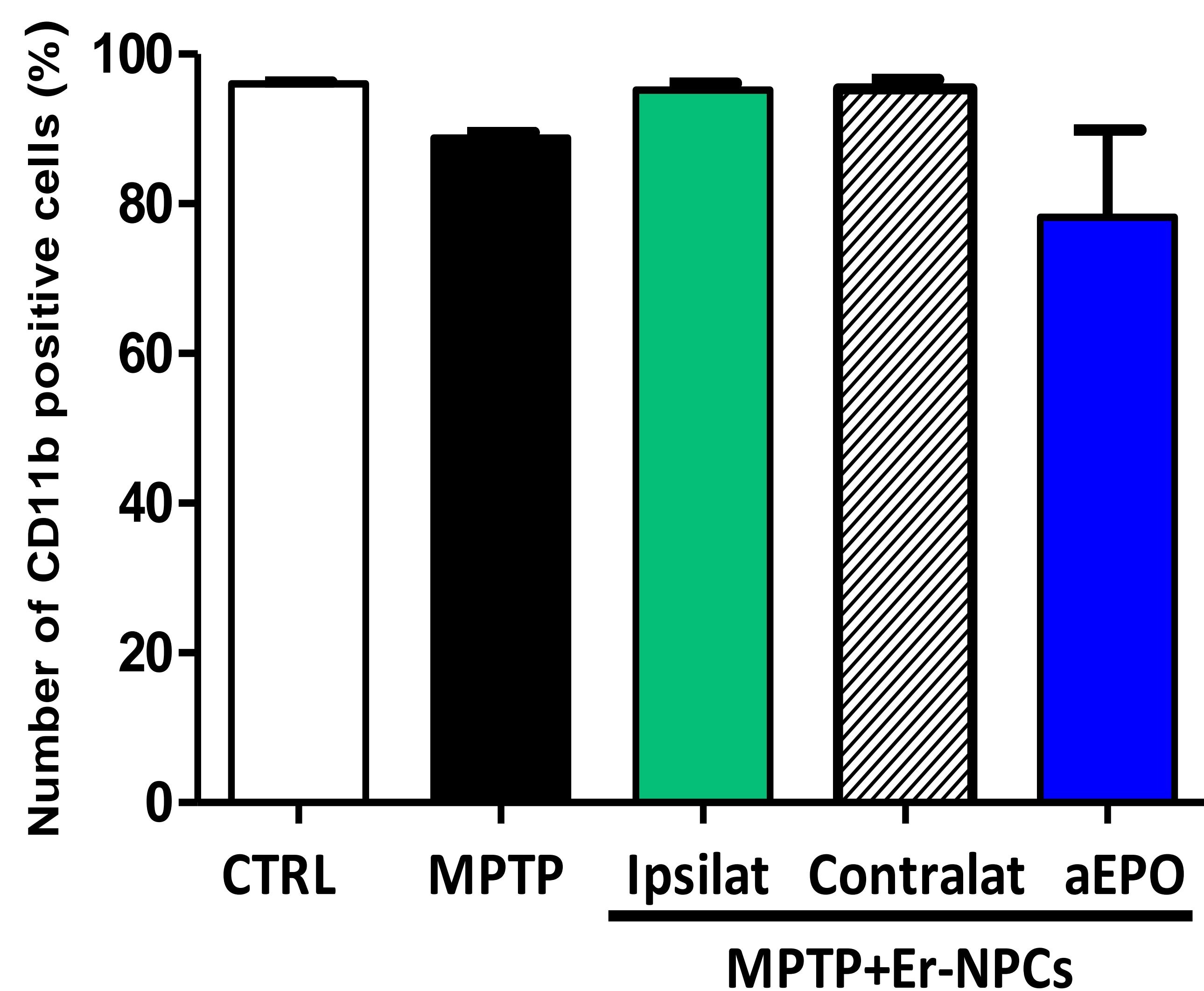

**B**

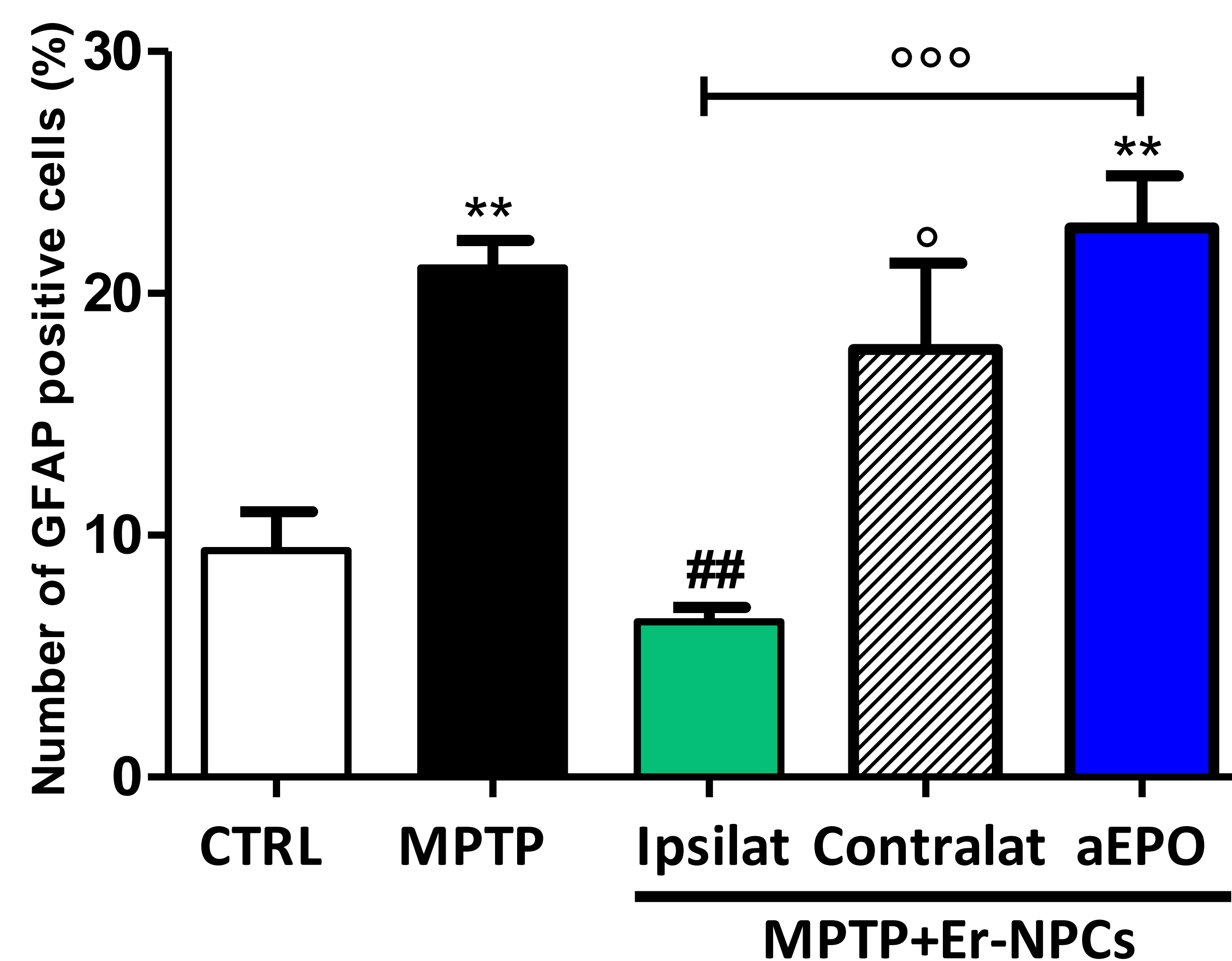

**C**

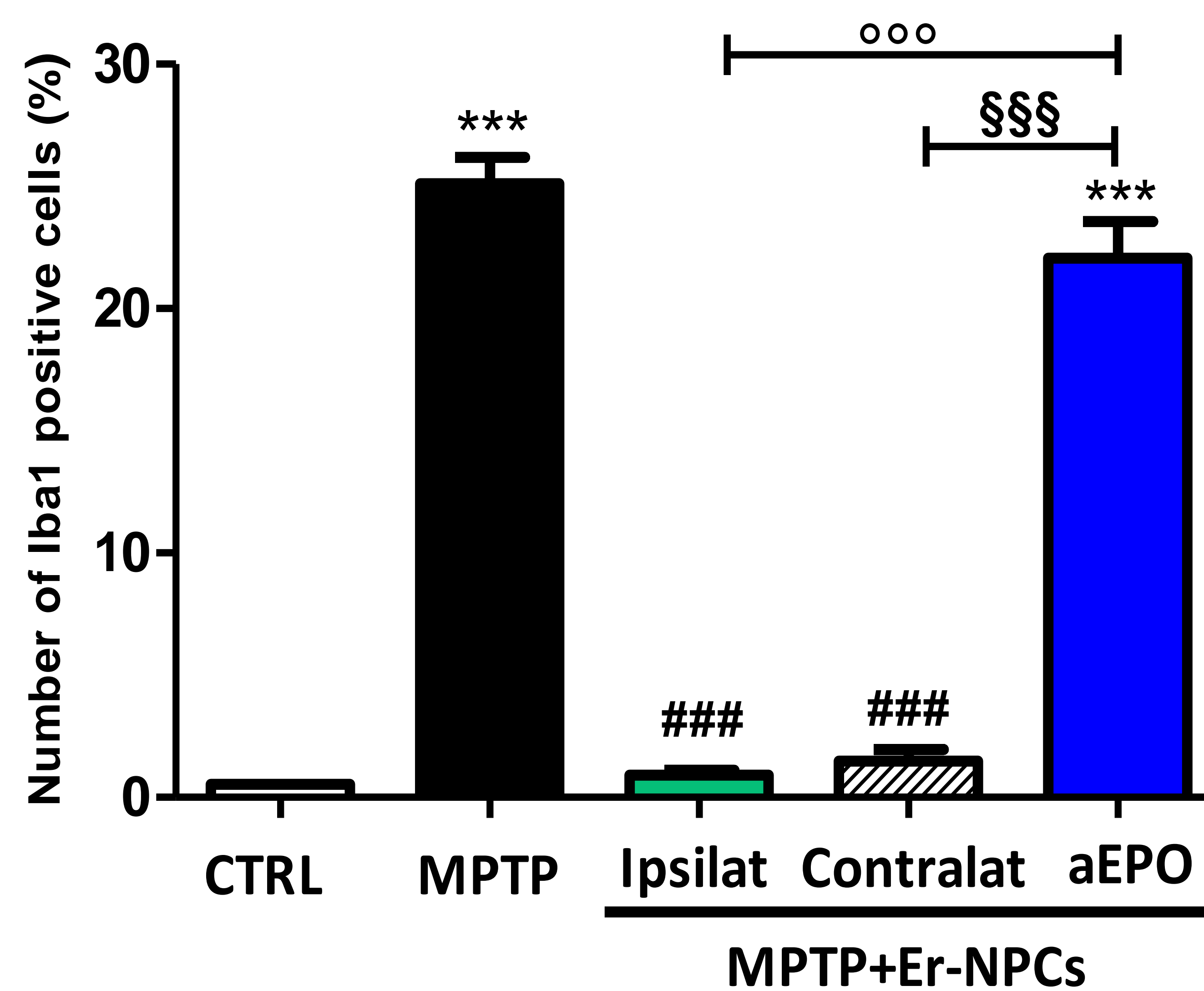

Supplement: Supplementary file 3 — Percentage of cells positive to CD11b, GFAP, and Iba1. Graphs report the stereologic counts of positive cells to investigated markers quantified in nine different fields for each condition (three mice for each group). The analysis was performed 2 weeks after Er-NPCs injection. Quantification was done by ImageJ picture analysis software. Data are expressed as mean ± SD. ***p < 0.001; **p < 0.01 vs CTRL; ###p < 0.001; ##p < 0.01 vs MPTP; °°°p < 0.001; °p < 0.05 vs MPTP + Er-NPCs ipsilateral; §§§p < 0.001 vs MPTP + Er-NPCs contralateral. (PDF 185 kb) [file 12974_2018_1375_MOESM3_ESM.pdf]

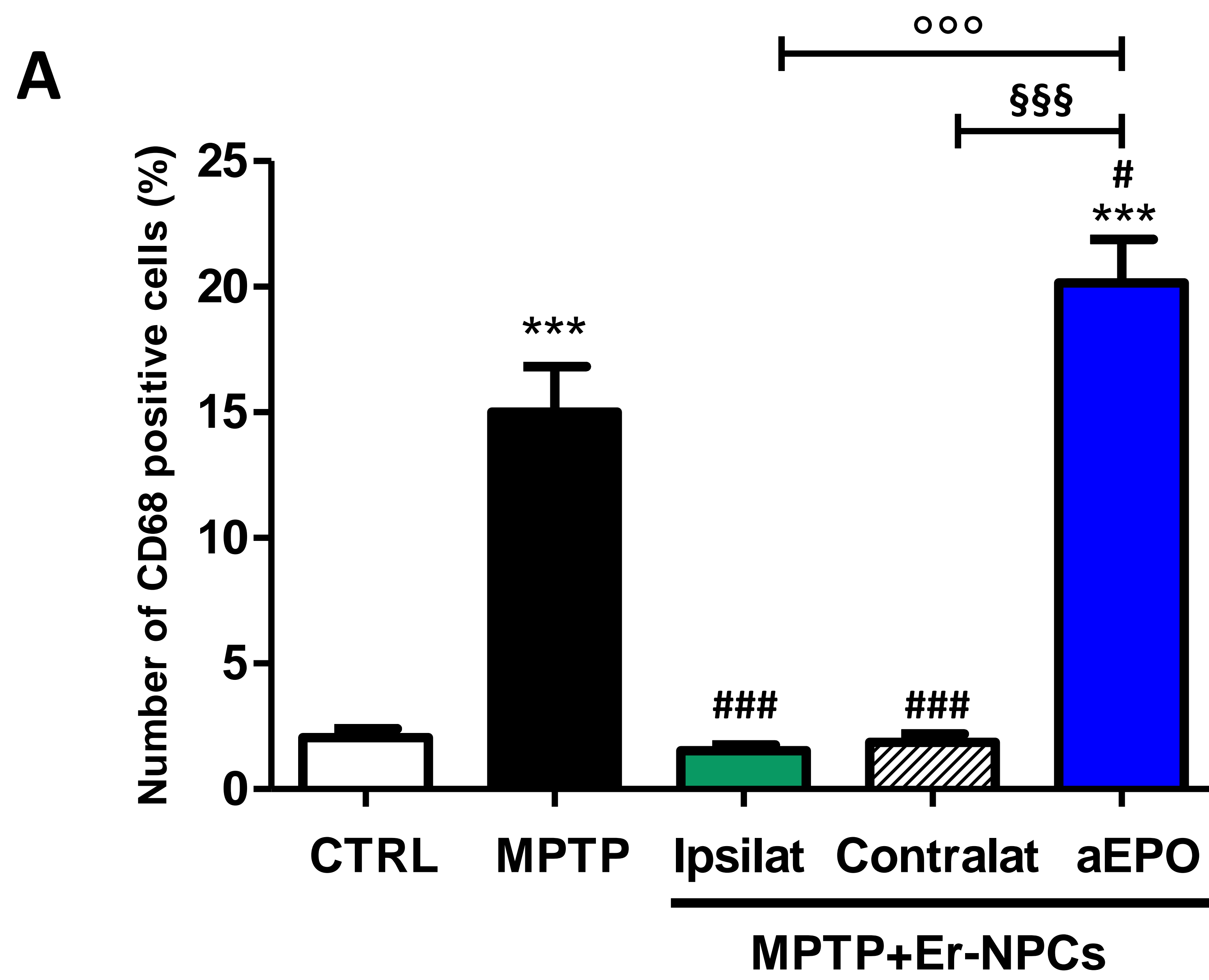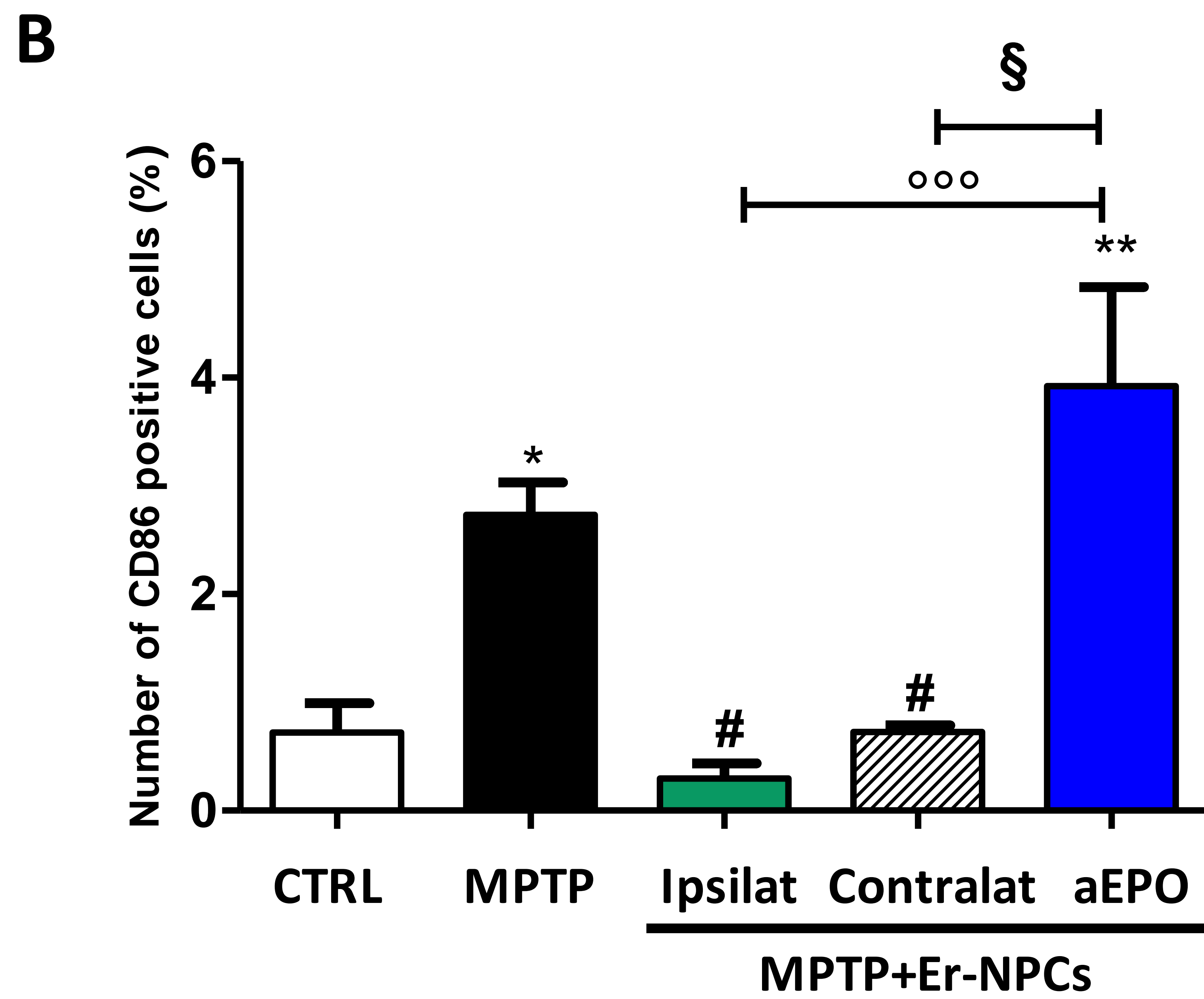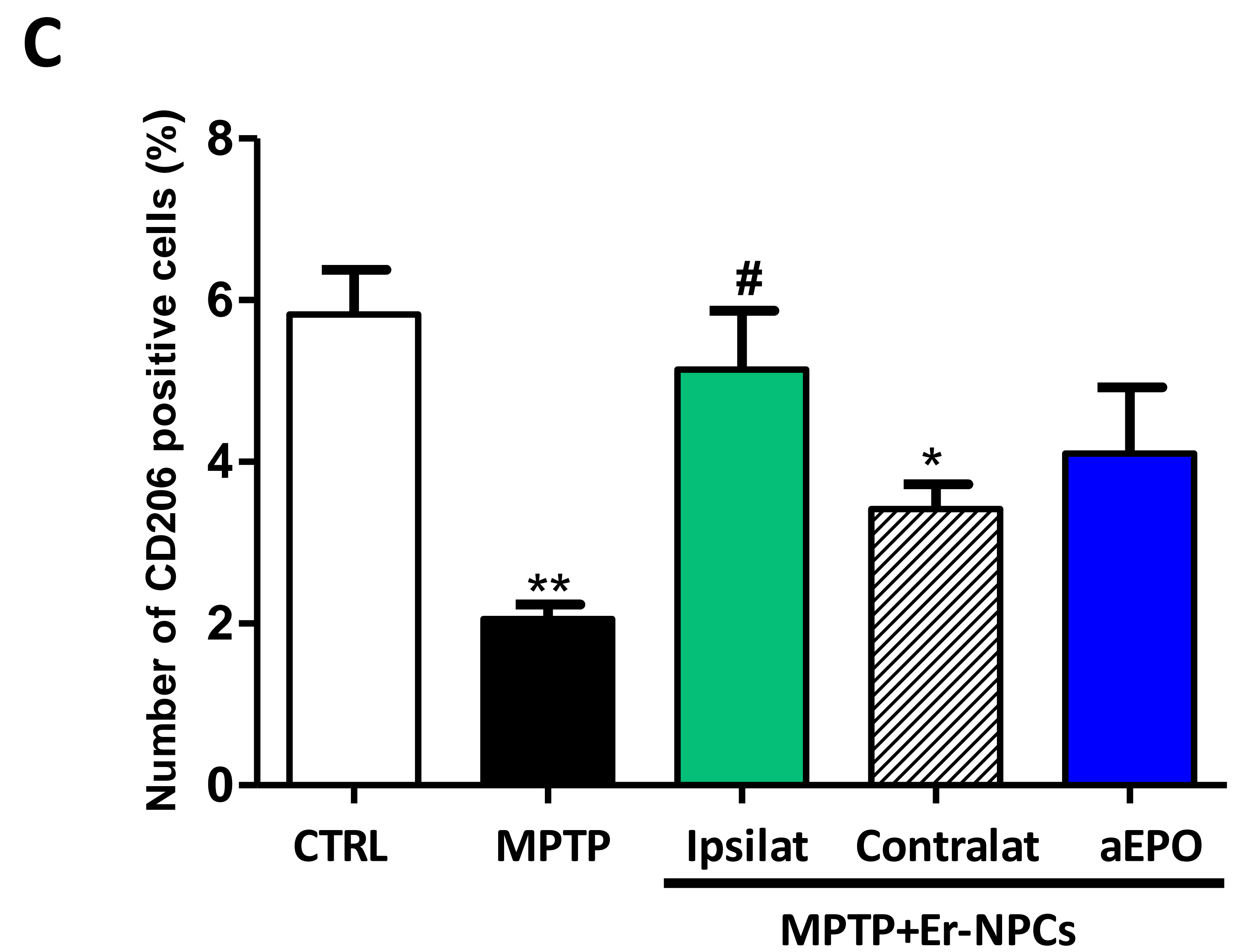

Supplement: Supplementary file 4 — Percentage of cells positive to CD68, CD86, and CD206. Graphs report the stereological counts of positive cells to the investigated markers quantified in nine different fields for each condition (three mice for each group). The analyses were performed by evaluating nine different fields for each condition (three mice for each group). Quantification was done by ImageJ picture analysis software. Data are expressed as mean ± SD. ***p < 0.001; **p < 0.01; *p < 0.05 vs CTRL; ###p < 0.001; #p < 0.05 vs MPTP; °°°p < 0.001 vs MPTP + Er-NPCs ipsilateral; §§§p < 0.001; §p < 0.05 vs MPTP + Er-NPCs contralateral. (PDF 181 kb) [file 12974_2018_1375_MOESM4_ESM.pdf]
